# The effect of marker types and density on genomic prediction and GWAS of key performance traits in tetraploid potato

**DOI:** 10.3389/fpls.2024.1340189

**Published:** 2024-03-08

**Authors:** Trine Aalborg, Elsa Sverrisdóttir, Heidi Thorgaard Kristensen, Kåre Lehmann Nielsen

**Affiliations:** Department of Chemistry and Bioscience, Aalborg University, Aalborg, Denmark

**Keywords:** *Solanum tuberosum*, genomic prediction, GBLUP, tetraploid potato breeding, GWAS, marker density, marker type

## Abstract

Genomic prediction and genome-wide association studies are becoming widely employed in potato key performance trait QTL identifications and to support potato breeding using genomic selection. Elite cultivars are tetraploid and highly heterozygous but also share many common ancestors and generation-spanning inbreeding events, resulting from the clonal propagation of potatoes through seed potatoes. Consequentially, many SNP markers are not in a 1:1 relationship with a single allele variant but shared over several alleles that might exert varying effects on a given trait. The impact of such redundant “diluted” predictors on the statistical models underpinning genome-wide association studies (GWAS) and genomic prediction has scarcely been evaluated despite the potential impact on model accuracy and performance. We evaluated the impact of marker location, marker type, and marker density on the genomic prediction and GWAS of five key performance traits in tetraploid potato (chipping quality, dry matter content, length/width ratio, senescence, and yield). A 762-offspring panel of a diallel cross of 18 elite cultivars was genotyped by sequencing, and markers were annotated according to a reference genome. Genomic prediction models (GBLUP) were trained on four marker subsets [non-synonymous (29,553 SNPs), synonymous (31,229), non-coding (32,388), and a combination], and robustness to marker reduction was investigated. Single-marker regression GWAS was performed for each trait and marker subset. The best cross-validated prediction correlation coefficients of 0.54, 0.75, 0.49, 0.35, and 0.28 were obtained for chipping quality, dry matter content, length/width ratio, senescence, and yield, respectively. The trait prediction abilities were similar across all marker types, with only non-synonymous variants improving yield predictive ability by 16%. Marker reduction response did not depend on marker type but rather on trait. Traits with high predictive abilities, e.g., dry matter content, reached a plateau using fewer markers than traits with intermediate-low correlations, such as yield. The predictions were unbiased across all traits, marker types, and all marker densities >100 SNPs. Our results suggest that using non-synonymous variants does not enhance the performance of genomic prediction of most traits. The major known QTLs were identified by GWAS and were reproducible across exonic and whole-genome variant sets for dry matter content, length/width ratio, and senescence. In contrast, minor QTL detection was marker type dependent.

## 1 Introduction

Since its original domestication from Peruvian wild species progenitors into Andean and Chilean landraces (Spooner et al., 2005, 2014), the cultivated potato has been globally disseminated, and *Solanum tuberosum L*. is currently the world’s third most important food crop (FAOSTAT, 2023). Its high efficiency in energy yield per cultivated area and high nutritional value compared to cereals have resulted in a cosmopolitan growth distribution (Wilson et al., 2021), and potato remains a crop of key interest for future global food security. However, to accommodate the expected 35%–56% increase in food demand by 2050 compared to 2010 (Van Dijk et al., 2021), as well as the exhaustion of fertile farmlands and imminent climate changes, it is paramount to intensify and accelerate the development of more sustainable crop strains (Lenaerts et al., 2019) with improved yield, pest resistance, and space/nutrient/water use efficiency.

The rapid progression of genome sequencing technologies and the availability of reference genomes for potato (The Potato Genome Sequencing Consortium, 2011; Sharma et al., 2013; Pham et al., 2020) have paved the way for the implementation of genome-assisted breeding methods, such as genomic selection (GS), in potato crop breeding (Hickey et al., 2017). Genomic selection breeding relies on a set of thousands of genome-wide markers, obtained by, e.g., single-nucleotide polymorphism (SNP) microarrays or genotyping by sequencing (GBS), and it is then assumed that all quantitative trait loci (QTL) are in linkage disequilibrium with at least one marker (Meuwissen et al., 2001). The marker effects on traits are then estimated using a model trained on a panel of genotypes and phenotypes, and the model is used to calculate the genomic estimated breeding values (GEBVs) from genotype data on a breeding population, facilitating directed parent crossing and early selection of breeding candidates without the need for direct phenotyping (Heffner et al., 2009). GS is particularly useful for traits with complex, multigenic inheritance patterns, such as chipping quality and dry matter content, where optimization has been largely ineffective in traditional breeding by phenotype-directed sexual crosses (Wilson et al., 2021). Several studies have already evaluated the performance of different statistical methods and machine learning approaches for the reliable prediction of genomic estimated breeding values for a host of agronomic performance traits, training on a vast collection of elite breeding materials (Slater et al., 2016; Sverrisdóttir et al., 2017, 2018; Stich and Van Inghelandt, 2018; Byrne et al., 2020; Selga et al., 2021; Wilson et al., 2021; Ortiz et al., 2022, 2023; Pandey et al., 2023). However, the development of strategies for enhancing the power of GS is still highly relevant for potato breeding.

While the crop fitness traits can have highly different genetic characteristics and heritabilities, all phenotypic variations are underpinned by causal alleles with either deleterious or advantageous trait effects under selection pressure, the former dominating in frequency in domesticated lineages (Zhu et al., 2022). In contrast to diploid crops, deleterious alleles that, e.g., induce frameshift mutations, create non-synonymous base changes, reform splice sites, or generate alternative stop codons, are ineffectively purged from breeding population gene pools during purifying selection due to the autotetraploid status of crop potato (Pham et al., 2017). In addition, insufficient recombination events in clonal propagation (Zhu et al., 2022) and the generally recessive nature of those alleles (Dwivedi et al., 2023) have led to a high abundance of deleterious alleles during domestication and clonal propagation, which is why elite potato, despite their high heterozygosity, exhibits signs of acute inbreeding depression (The Potato Genome Sequencing Consortium, 2011; Zhang et al., 2019). At the same time, the fixation of a few targeted desirable, recessive alleles in a single breeding line or population is extremely challenging in the case of tetrasomic inheritance (Muthoni et al., 2015). For breeding purposes, the identification of the subset of deleterious and beneficial alleles that manipulate phenotypic variation would, in principle, allow more accurate genomic prediction modeling based on the high information-carrying diagnostic markers alone (Zhang et al., 2018; Ramstein and Buckler, 2022). However, the complex multigenic signature and/or low heritability of several tuber quality traits and yield combined with the considerable environmental component of the observed phenotypes of such traits have complicated full genetic trait characterization (van Eck, 2007). GS models are instead traditionally based on a high density of anonymous genome-wide markers (Heffner et al., 2009), but the introduction of large amounts of redundant data has unknown consequences for model performance. An issue of similar concern arises for genome-wide association studies (GWAS). GWAS can be used to identify the subset of the total genetic variation that underpins target tuber and plant quality traits (Naeem et al., 2021). Elucidation of trait genetic architectures and trait-associated markers by this method as well as QTL mapping can be used to direct breeding efforts and selection models toward high-impact alleles, and several markers for the selection of tuber traits have already been identified (Fischer et al., 2013; D’hoop et al., 2014; Schreiber et al., 2014; Li et al., 2019; Byrne et al., 2020; Zia et al., 2020; Park et al., 2021; Ahmad et al., 2022). However, the sheer quantity of genetic variation in the potato genomes with, on average, one SNP per 29 bp (The Potato Genome Sequencing Consortium, 2011) represents a challenge. Though most variants will have a neutral effect and not impact trait phenotype, the inclusion of these observations results in reduced power of association due to increased stringency of correction for multiple testing but might not necessarily contribute to proportional amplifications of the QTL signal intensities.

Intuitively, reducing the background of redundant marker density by selecting only non-synonymous variants, which are expected to underpin the largest genetic contribution to phenotypic variance, can be expected to increase genomic prediction accuracy. While marker reduction to a set of non-synonymous variants is certain to reduce the stringency of correction in GWAS, it is also possible that including only functional effect variants will improve the signal power of additional minor effect QTLs by concentrating high-information variants. Following this hypothesis, we evaluated how GS model prediction accuracy and the resolution of GWAS were impacted by using different single-nucleotide polymorphism marker subsets: (i) amino acid-changing SNPs within protein-coding genes, non-synonymous SNPs (nsSNPs), (ii) amino acid-conserving SNPs within protein-coding genes, synonymous SNPs (sSNPs), (iii) SNPs located outside exons, non-coding SNPs (ncSNPs), and (iv) a combination of all types, as well as assessing the effect of marker reduction on GS models. We genotyped a panel of 762 clones called MASPOT by GBS, used in previous studies (Sverrisdóttir et al., 2017, 2018), and trained GBLUP models to predict GEBVs for five agronomic traits with different heritabilities and modes of inheritance, namely, chipping quality, dry matter content, yield, length/width ratio, and senescence using each of the filtered marker subsets in order to evaluate model response to marker type and robustness to marker reduction. We used single-trait GWAS to identify the associated loci for each of the five traits and assessed whether the information level of the trait genetic architecture carried by different functional variants differed.

## 2 Materials and methods

All statistical analyses and graphics were performed using R Statistical Software (v4.3.1) (R Core Team, 2023) in RStudio (v2023.6.2.561) (Posit team, 2023). Graphics were generated using the ggplot2 package (v3.4.3) in R (Wickham, 2016) unless otherwise stated.

### 2.1 Plant material

A mapping population called the MASPOT population, consisting of circa 5,000 offspring, was established at the LKF Vandel breeding station (presently Danespo A/S) in Vandel, Denmark. The MASPOT population was generated by the systematic cross-pollination of 18 elite potato cultivars in a full-diallel crossing design, the parents being either established cultivars or advanced breeding clones (Sverrisdóttir et al., 2017). The design was, however, limited by low fertility in specific crosses and male sterility in some of the parents. Male sterility is not an unusual trait in elite potato cultivars (Sanetomo and Gebhardt, 2015). A total of 762 clones were chosen randomly from the full mapping population and is henceforth referred to as the MASPOT panel in this paper (**Supplementary Figure 1**). The 762 offspring were planted in field trials in Vandel, Denmark, in 2013 and 2014 as described in Sverrisdóttir et al. (2017, 2018). The plants were grown to a plant density of approximately 40,000 plants/hectare with 30 cm between plants and 75 cm between rows. In 2013, the tuber seedlings were planted in April 24 and 25 (no replicates) in 24-parcel blocks and harvested in August 11–29 (109–128 days after planting). The plants were desiccated 1 to 2 weeks before harvest. No checks were used. In 2014, the clones were divided into four groups based on parent earliness and planted in a randomized 28-parcel block design with two replicates. The groups were planted in April 24, 25, 28, or 29 and harvested in August 11–29 (109–129 days after planting), also with 1 to 2 weeks of desiccation. The groups were harvested in chronological order. A total of 19 checks were planted in two replicates, 18 of which were the MASPOT panel parents. The checks were inspected manually for signs of unusual development/disease infection. No abnormalities were observed, taken as an indication of credible plant material for all clones. As the population was highly diverse, not all plants had fully matured at harvest. The soil type was sandy loam. Fertilization was performed with 1,000 kg/hectare NPK 14-3-15. Pests and diseases were controlled with Fenix and Titus (weed) before and immediately after sprouting, Mospilan (insects) ultimo June and again ultimo July, and alternating Ranman and Revus (late blight) from approximately June 23 until desiccation as needed, depending on the weather. The fields were irrigated as needed. Additional details about the population can be found in Sverrisdóttir et al. (2017). Since the propagation of the population happened simultaneously with the trials, the number of seed tubers available in 2013 were lower than in 2014. Therefore, we have not used a formally established incomplete block design. Furthermore, the trial data is not corrected for soil heterogeneity due to the following reasons: (i) Danish regulations for crop rotation do not allow growing potatoes at the same site in two consecutive years and (ii) since significant senescence variation was observed in 2013, the plants were grouped by senescence in 2014 to minimize “neighbor vigor effects” in the trials. As a consequence, the data could be corrected reliably for soil heterogeneity.

### 2.2 Phenotyping and adjustment for environmental effects

Phenotyping of dry matter content and chipping quality is described in Sverrisdóttir et al. (2017). In short, dry matter content [%] was determined for the MASPOT panel and parent clones harvested in 2013 (one replicate) and 2014 (two replicates). The tubers were washed, and a basket holding 1.5–10 kg of tubers was weighed above and under water shortly after harvesting. The dry matter content was then calculated using the following empirical equation:


$$DM[\%]=214\cdot ((\frac{weight\;in\;air}{\left(weight\;in\;air)-(weight\;in\;water\right)})-0.988)$$

Chipping quality was determined as the chip color following frying in oil after the cold storage of tubers. Phenotyping of chipping quality was performed only for the 2013 harvested clones. The tubers were stored at either 4°C for roughly 2 months, after which they were incubated at ambient temperature 2–6 h prior to frying. Four to six slices (1 to 2 mm) of each tuber were fried in sunflower oil at 180°C until the bubbles ceased to emerge (generally 2 to 3 min). The frying color was visually assessed to a standard set on an arbitrary grading scale from 1 (dark) to 9 (light).

Yield was measured for the 2014 harvested clones in the field at harvest as the total weight of five tubers from each clone in two replicates, i.e., 2 × 5 tubers each. The weight was converted into hkg/ha values, assuming 40,000 plants/hectare to account for plot variations from year to year.

Length/width ratio was determined as the length/width ratio (LW) for the 2014 harvested clones:


$$LW=\frac{length}{width}$$

Tuber length was defined as the longest measure and the width as the measure perpendicular to this and measured on a SCOUT camera (Newtec A/S, No. 0213). The measures do not consider tuber anatomy, where length is defined as the distance from the rose (apex) to the heel (attachment of stolon). The true definition will only be violated for irregular tubers, which are relatively rare and hence assumed to not significantly affect the downstream analyses. Outliers were identified on the length or width (diameter) parameter relative to the nearest neighbor and removed following Dixon’s Q test (Dean and Dixon, 1951) before the calculation of length/width ratio, where the critical confidence level, *Q_crit_*, was estimated for batches of up to 200 tubers by regression and used for a two-tailed test as outlined by Rorabacher (1991).

Senescence (an earliness proxy) was scored manually on a scale from 9 (late senescence) to 1 (no senescence). Phenotyping was performed for the 2013 and 2014 harvested clones. The scoring was performed temporally at three points, splitting the scale accordingly: the first scoring (when the first cultivars begin dying off) used the upper end of the scale (9-8-7), the second used the middle of the scale (6-5-4), and the third scoring, capturing cultivars that display late senescence, used the lower end of the scale (3-2-1). Each clone was only scored once.

All phenotypic data were corrected for variation across years by fitting a linear mixed model to the phenotypic data *via* restricted maximum likelihood (REML) using the following model:


$$y_{ij}=µ+\;genotype_{i}+\;year_{j}+\;e_{ij}$$

where *y_ij_* is the observed phenotype, *μ* is the overall mean, *genotype_i_* is the random effect of the *i*th genotype, *year_j_* is the fixed effect of the *j*th year, and *e_ij_* is the error term (Sverrisdóttir et al., 2017). The model was made with the lme4 package in R (Bates et al., 2015). Terms for genotype-by-environment (G × E) were not included in either the GS or GWAS models since the experimental design did not produce sufficiently robust phenotyping to allow the rigorous estimation of this in the MASPOT panel (Sverrisdóttir et al., 2017, 2018), which is why the model was simplified to avoid infusion of additional error and lessen the risk of overfitting.

### 2.3 Genotyping

Genotyping was performed by GBS. GBS libraries were prepared according to Sverrisdóttir et al. (2017), following a protocol adapted from Elshire et al. (2011). The 5′ and 3′ adapters for Illumina sequencing were designed for a 96-multiplexing system. DNA was extracted from leaf tissue and digested with *ApeKI*. The fragments were ligated to adapters, pooled in 96-plex libraries, purified, and amplified by PCR. The MASPOT panel libraries were sequenced on a HiSeq 2000 (Illumina, San Diego, CA, USA) with single-read sequencing (100 bp), and each 96-plex library was sequenced on three channels on a flow cell.

### 2.4 Filtering raw sequence data, mapping, and SNP calling

Sequenced reads were processed as described in Sverrisdóttir et al. (2017). The reads were demultiplexed, trimmed, and mapped onto the *S. tuberosum* Group Phureja reference genome sequence [DM v4.03; (Sharma et al., 2013)]. SNPs were called using the Genome Analysis Toolkits (McKenna et al., 2010) UnifiedGenotyper tool with ploidy set to 4 and the minimum phred-scaled confidence threshold of 50 for variant calling and of 20 for variant omission (and filtered with LowQual if less than the calling threshold), as described in Sverrisdóttir et al. (2017). The SNPs were then filtered to a root mean squared quality of 30, including only biallelic variants. Since potatoes are not tetraploid across all loci, but rather have a mean gene copy number of 3.2 (Sun et al., 2022), enforcing the expectation of tetraploidy across all marker sites would constitute a confounding error. As a consequence, the called tetraploid genotypes were not used explicitly, but rather variant allele frequencies estimated from the sequencing data for each variant were used directly as genotypes for statistical analyses cf. (Ashraf et al., 2016) to accommodate the gene copy number variation across the potato genome. Minor allele frequency (MAF) was calculated from read coverage, and SNPs were filtered to a MAF of 1% (average variant frequency<0.99 and >0.01), a read coverage >5, and a missing rate of maximum 50%.

### 2.5 Marker reduction and filtering

SNPs were annotated using SnpEff (Cingolani et al., 2012) using a custom database built from the *S. tuberosum* DM v4.03 reference genome. The SNPs were filtered into three subsets based on annotation: non-synonymous (including missense, stop codon gain/loss, start codon gain/loss, frameshift, and exon loss variants), synonymous (including synonymous variants), and non-coding (excluding all exonic variants, reduced to every third non-coding variant), in addition to a combination set of the former three subsets. For GWAS, a 1-in-3 reduction of the combination set was also analyzed. For each of the four annotated SNP sets, the SNPs were further filtered to read coverage between 5 and 60, while individuals with >70% missing data were removed.

Reduced marker sets, for the evaluation of genomic prediction model tolerance to marker reduction, were prepared for each of the four SNP sets by iteratively reducing the sets to every other position, resulting initially in two bins. This was done to ensure whole-genome dispersion of the SNPs and avoid the introduction of regional bias. Following the second iteration, resulting in four bins, only four bins were kept for each iteration. Iterations were performed this way until ~150 markers remained in each SNP set. A final reduction to every 10th marker was then performed on each set. **Supplementary Figure 2** presents an overview of the marker reduction strategy. The SNP density plots were generated using the CMplot package in R (LiLin-Yin, 2023).

### 2.6 Statistical analyses

#### 2.6.1 Assessment of population structure

The population structure of the MASPOT panel was ascertained by performing a principal component analysis (PCA) using the prcomp function of the built-in stats package (R Core Team, 2023) on the genomic relationship matrix (**G**) computed from the combination set of annotated SNPs (93,170 SNPs) (**Supplementary Figure 3**). The genomic relationship matrix was created from the genotype matrix (**Z**) based on the first VanRaden (2008) method. The **Z** matrix contains the genotypes taken as allele frequencies for each sample and SNP from sequence data (Ashraf et al., 2016). The allele frequencies were calculated as the ratio between allele counts of the alternative allele and the total allele count, producing a value between 0 and 1. This allows the genotype matrix to capture tetraploid allele dosages.


$$AF\;=\;\frac{AC_{alt}}{AC_{ref}+AC_{alt}}$$

The allele frequencies were corrected for missing data following the correction, *w_i_*, described by VanRaden (2008):


$$w_{i}=\sqrt{\frac{\sum p_{k}(1-p_{k})\;over\;all\;loci}{\sum p_{k}(1-p_{k})\;\;over\;only\;non-missing\;loci}}$$

where *p_k_* is the mean allele frequency at locus *k*. A total of 16.26% of all markers were imputed. The genotype matrix was centered and adjusted for missing values according to Ashraf et al. (2016), whereafter means were set to zero, corresponding to mean imputation for missing data.


$$Z_{ik}=(X_{ik}-p_{k})\cdot w_{i}$$

where ***X*** *_ik_* is the allele frequency in family *i* at locus *k*. The genomic relationship matrix was computed from **Z** using global scaling, following method 1 of VanRaden (2008), with a modification to adjust for tetraploidy (Ashraf et al., 2014, 2016).


$$G=\frac{ZZ'}{0.25\sum p_{k}(1-p_{k})}$$

where $0.25\sum p_{k}(1-p_{k})$ is the sum of genotypic variance and also the average diagonal of ***ZZ*** '.

#### 2.6.2 Genomic prediction models

Genomic predictions for each of the single traits, using each of the generated SNP sets, were performed using a standard additive GBLUP model, equivalent to a ridge-regression with uniform shrinkage of SNP effects, without accounting for marker effect size, i.e., assuming that each marker accounts for an equal proportion of the total genetic variance, though shrinkage is dependent on sample size and allele frequency (Gianola, 2013). A G × E term was not included due to insufficient robustness of across-year phenotyping (Sverrisdóttir et al., 2017). GEBVs are directly estimated using the genomic relationship matrix (Meuwissen et al., 2001):


y=1μ+g+e

where ***y*** is a vector of observed phenotypes, *µ* is the mean, ***e*** is a vector of residual effects with $e\sim N(0,\;I\sigma_{e}^{2})$ , where **I** is an identity matrix and $\sigma_{e}^{2}$ is the residual variance, and ***g*** is a vector of random genomic breeding values with distribution $g\sim N(0,G\sigma_{g}^{2})$ is the genetic variance of the model. All models were computed using the BGLR package in R (Pérez and de los Campos, 2014) with default settings for priors and settings of 12,000 iterations and a burn-in of 2000. All analyses were performed using an eightfold cross-validation scheme, where clones were randomly divided into eight groups, one group being used for validation while the model was trained using the data of the seven remaining groups. This process was repeated, each time with a different group as validation set, until predictions had been calculated for all individuals. Each analysis was repeated with 10 different cross-validation groupings, and the GEBV was calculated as the average across all samplings. The accuracy of the GEBVs was determined as the Pearson correlation coefficient between the predicted GEBVs and the observed phenotypes, described here as the prediction correlation:


r(GEBV:y)

Correlation coefficients for each trait, when using ~30k markers, were compared pairwise by Welch two-sample *t*-test with Bonferroni correction of significance level to 0.05/*N*, where *N* is the total number of tests for each trait. A linear regression of the observed phenotypes on the predicted values was used as a measure of bias of the GEBVs, where a regression slope of *β*=1 indicates no bias, *β*<1 implies that extremely high (low) GEBVs over-(under)estimate the observed phenotype and *vice versa* for *β*>1 (Luan et al., 2009). The prediction correlation and bias summary statistics were evaluated for all modeled SNP sets.

#### 2.6.3 Heritability

The pedigree narrow sense heritabilities ( $h_{p}^{2}$ ) were estimated for each trait as the linear regression coefficient of the mid-parent phenotypic value (i.e., mean parental phenotype) against the offspring value. The offspring of one or more parent with missing phenotypic data was not included. Genomic narrow sense heritability was estimated as the ratio of genomic to phenotypic variance using the genomic relationship matrix of the full combination data set in a REML analysis (de los Campos et al., 2015).


$$h_{g}^{2}=\frac{\sigma_{g}^{2}}{\sigma_{y}^{2}}$$

#### 2.6.4 Genome-wide association studies

Genome-wide association studies were conducted for each of the four fully annotated SNP sets. Additionally, for the combination SNP set, a reduced dataset was prepared by taking every third SNP, generating a reduced set of 31,032 SNPs, for which GWAS was also conducted. GWAS was performed by single marker regression with the regress package in R (Clifford and McCullagh, 2006, 2020) using the following model for each SNP in the respective data subsets, cumulating the marker effects:


$$y=1\mu +X_{i}\beta_{i}+g+e$$

where ***y*** is a vector of observed phenotypes, *µ* is the mean, ***X_i_*** is the vector of SNP genotype values taken as allele frequency at the *i*th position, *β_i_* is the corresponding additive genetic effect of the *i*th SNP, ***g*** is a vector of random genomic breeding values with distribution $g\sim N(0,G\sigma_{g}^{2})$ , where ***G*** is the genomic relationship matrix of Sverrisdóttir et al. (2017) computed for the MASPOT panel, and each SNP set, $\sigma_{g}^{2}$ , is the genetic variance of the model, and ***e*** is a vector of residual effects with $e\;\sim \;N(0,\;I\sigma_{e}^{2})$ . The genomic relationship matrix was included as a fixed effect in the model to correct for the genomic relationship between the MASPOT offspring. For each chromosome, a **G**-matrix was calculated based on the SNPs mapped to the remaining chromosomes, excluding the target chromosome. This **G**-matrix was then used to correct for population structure for the SNPs of the excluded chromosome to ensure that SNPs were not included in the model twice (Kristensen et al., 2018).

Potatoes are clonally propagated, and modern cultivars can be expected to have a significant proportion of recurrent genetic variation in their pedigree. This leads to a population structure which, in turn, has a tendency to cause overdispersion of the test statistics in association analyses (Devlin et al., 2001). This would result in an increased number of false positive associations. To adjust for this, we calculated genomic inflation factors, *λ_gc_*, for each trait and SNP subset and used to correct *p*-values for inflation due to systematic effects not captured by the model according to Hinrichs et al., (2009) and Kristensen et al. (2018). Inflation factors were computed as the median value of the chi-squared statistic of the SNPs divided by the expected median value, i.e., assuming no association between the SNPs and the trait.


$$\chi^{2}=Q_{\chi^{2}}^{-1}(P,\;1)$$

$$\lambda_{gc}=\frac{median(\chi^{2})}{Q_{\chi^{2}}^{-1}(0.5,\;1)}$$

Each *p*-value (*P*) is converted to a *χ* ^2^ quantile using the quantile function of the chi-squared distribution, i.e., the inverse of the cumulative distribution function (CDF) $Q_{\chi^{2}}$ , with one degree of freedom. To determine the genomic inflation factor, *λ_gc_*, the median of the *χ* ^2^-quantiles is then divided with the chi-square of the 50th percentile with one degree of freedom, i.e., the expected median *χ* ^2^, assuming no SNP–trait association.

For *λ_gc_* >1, the chi-squared quantile of the *p*-values was divided by the inflation factor and used to calculate corrected *p*-values using the CDF of the chi-squared distribution with one degree of freedom.


$$P_{corrected}=1-Q_{\chi^{2}}((\frac{\chi^{2}}{\lambda_{gc}}),1)$$

To control false positive associations, Bonferroni correction was used with a false discovery rate of p<0.05/N , where 0.05 is the overall significance threshold and *N* is the total number of markers tested in the analysis. The proportion of phenotypic variance explained by markers was calculated using the formula from Shim et al. (2015). QQ and Manhattan plots were plotted using the qqman package in R (Turner, 2018).

## 3 Results

### 3.1 Genotyping statistics

Sequencing yielded an average of four million trimmed and filtered reads per sample for the MASPOT panel of 762 clones. A total of 3.4 million variant sites were found. Following filtering for MAF > 1%, minimum coverage of five, and a missing rate of maximum 50%, 182,757 variants remained with sequence positions as in Sverrisdóttir et al. (2017).

#### 3.1.1 Filtering and reduction of markers according to SNP annotation

Four subsets of SNP data were created from the annotation filter: (1) non-synonymous variants (32,352 nsSNPs), synonymous (34,695 sSNPs), non-coding (33,743 ncSNPs), and a combination set (100,790 of all SNPs). After filtering each data set to read coverage between 5 and 60 and individuals with >70% missing data, the data sets were reduced as presented in **Table 1** (**Supplementary Files 1–4**).

**Table 1:** Number of single-nucleotide polymorphisms (SNPs) and individuals in each data set.

SNP set	SNPs after first filtering	SNPs after second filtering	Individuals after filtering
	MAF > 1%, coverage > 5, missing rate<50%	5< coverage <60, missing rate<70%	MASPOT panel
Full	182,757	171,859	755
Non-synonymous	32,352	29,553	755
Synonymous	34,695	31,229	755
Non-coding	33,743	32,388	751
Combination (non-synonymous, synonymous, non-coding)	100,790	93,170	755

Total number of individuals before filtering: 762 in MASPOT panel.

**Figure 1:**
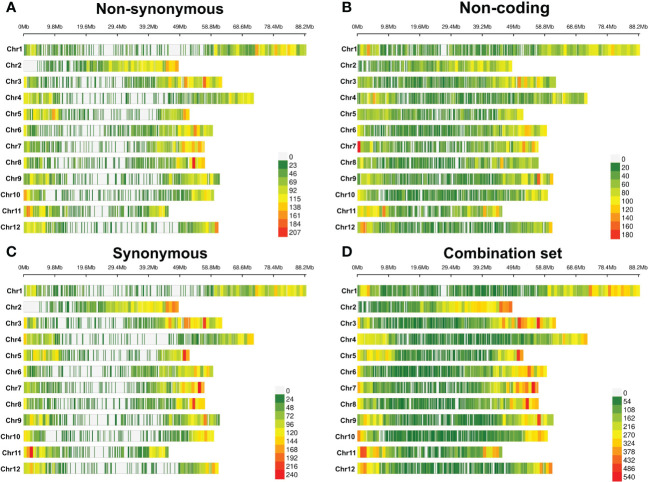
Heat map of marker density in 1-Mb windows for each chromosome. **(A)** Non-synonymous variants, **(B)** non-coding variants, **(C)** synonymous variants, and **(D)** combination set of annotated variants. The color gradient denotes marker count.

Markers were well distributed over each of the 12 chromosomes in each of the four annotation-based data sets (**Figure 1**, **Supplementary File 5**), consistent with marker density distributions found in another study of tetraploid potato (Wilson et al., 2021). The markers most densely populated the apocentromeric regions (The Potato Genome Sequencing Consortium, 2011) in all sets, with the two exonic variant sets presenting with Mb-sized windows of SNP sparsity in and around the centromeres. This is consistent with low gene density (Sun et al., 2022) and repressed meiotic recombination (Marand et al., 2017) in the pericentromeric regions of potato genomes, leading to reduced polymorphisms, and hence marker density as well as enrichment of fixed deleterious alleles (Zhang et al., 2019), and tight genetic linkage in these genomic areas.

### 3.2 Phenotypes and trait heritability estimates

**Figure 2:**
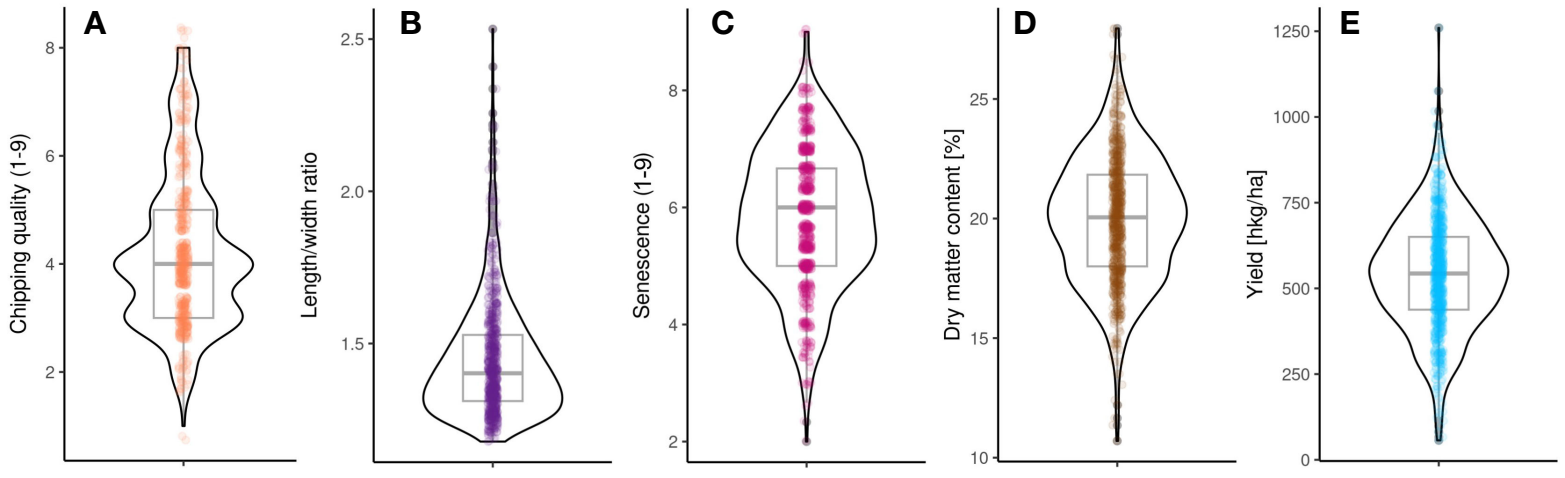
Distribution of phenotypes in the MASPOT panel. **(A)** Chipping quality, **(B)** length/width ratio, **(C)** senescence, **(D)** dry matter content, and **(E)** yield.

Phenotypes for yield, dry matter content, chipping quality, length/width ratio, and senescence (a proxy of earliness) were assessed for the 755 individuals retained in the MASPOT panel after full SNP data filtering (**Figure 2**, **Supplementary File 6**). The phenotypes were corrected for yearly effects between seasons using a linear mixed model only, as our data did not allow the rigorous estimation of G × E effects (Sverrisdóttir et al., 2017). Chipping quality phenotypes were missing for 31% of the MASPOT panel. Following correction, the phenotypic data appeared approximately normally distributed for all traits, except for length/width ratio, following a quotient distribution, and chipping quality (**Supplementary Figure 4**), presenting with a right skew. This was most pronounced for length/width ratio and was likely a result of this being a ratio distribution of two normally distributed variables (length and width, respectively) (Díaz-Francés and Rubio, 2013). Regardless, all phenotypes were used for GS and GWAS models without transformation, and the significance threshold indicator lines used in Manhattan plots were based on Bonferroni correction for all phenotypes.

Pedigree heritabilities (**Supplementary Figure 5**) were generally estimated as higher compared to genomic narrow sense heritability (**Table 2**). Yield exhibited the lowest heritability, with genomic and pedigree heritabilities of 22% and 30%, respectively, while dry matter content had the highest pedigree heritability of 91%, but only 41% genomic heritability. The difference in genomic narrow sense heritabilities may be a result of insufficient sampling of the true genomic diversity in the 93,170 marker combination set to accurately estimate relatedness as suggested in Sverrisdóttir et al. (2017). However, the specific large difference for dry matter content cannot be explained by such a general effect. We speculated that this may be related to the fact that the cytoplasmic type of potato. cytoplasmic type W/γ, is positively correlated with starch content (Sanetomo and Gebhardt, 2015) and therefore dry matter content. The cytoplasmic type is captured in the pedigree heritability estimates, but since genomic markers are derived from nuclear DNA, this is likely not captured in the genomic heritability estimate. The means and median dry matter contents of offspring of W/γ-type cytoplasm mother (male-sterile) were compared with the remaining panel. Indeed they were significantly different (*p* = 2.2*10^-16^), indicating that the cytoplasmic markers related to male sterility, not captured in the genomic markers, could constitute some/all of the unaccounted genetic diversity underpinning the trait heritability. However, removing W/γ individuals and parents from the analysis, the pedigree heritability was 95% and the genomic estimates 40%, in essence the same values as obtained with the entire panel.

**Table 2:** Mean, range, phenotypic and genetic variance, coefficient of variance (CV), genomic narrow sense heritability, and pedigree narrow sense heritability.

Phenotype	Mean	Range	Phenotypic variance	Genetic variance	Phenotypic CV (%)	Genetic CV (%)	$h_{p}^{2}$ (%)	$h_{g}^{2}$ (%)
Chipping quality	4.24	1–8	2.02	1.25	98.44	77.34	74.02	61.73
Length/width ratio	1.45	1.18–2.53	0.04	0.02	3.33	2.25	59.01	45.56
Senescence	5.83	2–9	1.41	0.53	20.33	12.47	60.55	37.61
Dry matter content	19.98	10.7–27.95	6.89	2.80	13.14	8.37	91.17	40.55
Yield	540.81	56.73–1,259.68	26,528.26	5,710.41	30.12	13.97	29.71	21.53

### 3.3 Population structure characterization

**Figure 3:**
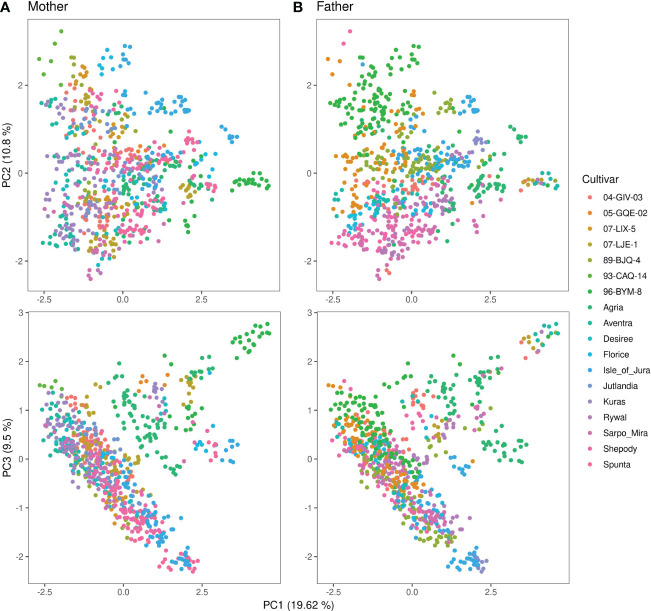
Principal component analysis of the genomic relationship matrix of the annotated combination set of 93,170 SNP markers for 755 MASPOT panel clones colored by **(A)** mother and **(B)** father. Principal component 1 is plotted against principal component 2 in the top and against principal component 3 in the bottom. The principal components explain 19.62%, 10.8%, and 9.5% of the total explained variance, respectively.

**Figure 3** shows the first three principal components from the PCA of the genomic relationship matrix of the 93,170 marker annotated combination data set. The offspring of the full diallel cross showed no clear separation into distinct genetic groupings based on PC1 and PC2, though same-father siblings generally congregated closer within the span of the full panel. This trend was less pronounced for same-mother siblings. Plotting PC1 against PC3 showed some offspring diverging genetically from the main group. These individuals were generally progeny of 93-CAQ-14 (father), Agria (mother), or 96-BYM-8 (mother) [Agria grandmother], in addition to a few clones with miscellaneous parentage. Beyond 96-BYM-8 being a descendent of Agria, their pedigrees do not allude to any distinct features of these MASPOT parents’ heritages (data not shown). The population structure is corrected for in both GS and GWAS through the **G**-matrix.

### 3.4 Genomic prediction

#### 3.4.1 Effect of marker filtering and density

**Figure 4:**
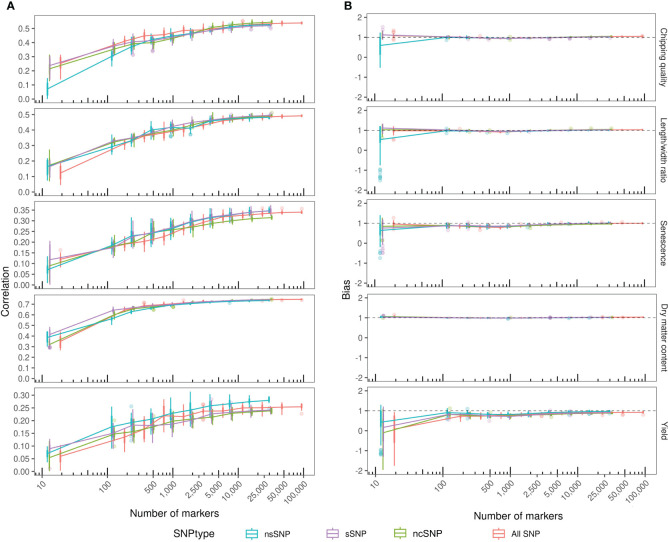
GBLUP prediction correlation coefficient and bias within the MASPOT panel over a number of annotated markers. The markers were reduced from full annotated SNP data sets by iterative reduction to every other marker (every 10th for the final reduction) to avoid the introduction of positional bias. Each reduction was repeated up to four times for each iteration. Color by marker annotation (SNPtype). **(A)** Prediction correlation coefficient between observed and predicted phenotypic values. Boxplots of correlation coefficients determined for each marker count with connecting lines through the mean. **(B)** Bias of GEBVs estimated as the slope of the linear regression line between observed and predicted phenotypic values. Boxplots of biases determined for each marker count with connecting lines through the mean bias.

Genomic predictions with marker reduction were conducted based on the four annotated subsets, non-synonymous, synonymous, non-coding, and the combination set. The correlations between observed phenotypes and GEBVs calculated for each individual using GBLUP models with eightfold cross-validation and 10 repeats are shown in **Figure 4A**. The highest correlation was obtained for dry matter content of 0.75 using the ncSNPs (**Table 3**). However, performance was not significantly different from sSNPs and the combined SNP set. Only the nsSNPs produced a significantly poorer prediction accuracy than the other three sets (*p* = 7.9*10^-5^ vs. sSNP, 5.0*10^-6^ vs. ncSNPs, and 2.0*10^-5^ vs. all SNPs), but still yielding a mean correlation coefficient of 0.74. In all dry matter content cases, the prediction accuracies plateaued at around 1,000 markers. Chipping quality and length/width ratio could be modeled to intermediate correlations of maximum 0.54 and 0.50, respectively. For chipping quality, filtering the markers to nsSNPs and sSNPs produced significantly lowered prediction accuracies compared to using ncSNPs alone (*p* = 6.2*10^-4^ and 4.1*10^-5^, respectively) or a combination of all SNPs for over 25,000 markers (*p* = 6.3*10^-3^ and 3.5*10^-4^), while the correlation coefficients plateaued at ~10,000 markers. The numerical difference in performance was, however, only slight. For length/width ratio, only sSNPs performed significantly better than nsSNPs (0.50 compared to 0.48, *p* = 3.3*10^-3^), while no statistically significant difference could be observed between the remaining data sets for over 25,000 markers. Traits senescence and yield had the lowest correlation coefficients of maximum 0.35 and 0.28, respectively, and the correlation coefficients did not reach a plateau for other than the combination set when using ~50,000 markers. For senescence, there was not a statistically significant best-performing annotation set—only the ncSNP models gave a statistically significant worse prediction accuracy >25,000 SNPs, with a mean correlation coefficient of 0.32 (*p* = 4.9*10^-6^ vs. nsSNPs, 5.1*10^-5^ vs. sSNPs, and 7.3*10^-7^ vs. all SNPs). For modeling yield, the nsSNPs produced significantly best results, improving correlation coefficients by 0.03–0.04 compared to the other SNP types when using >25,000 markers (*p* = 2.8*10^-6^ vs. sSNP, 9.1*10^-7^ vs. ncSNPs, and 9.0*10^-5^ vs. all SNPs).

**Table 3:** Mean prediction correlation ± standard deviation observed for each trait across all annotation datasets using ~30,000 markers for modeling.

Trait	Non-synonymous SNPs (29,553)	Synonymous SNPs (31,229)	Non-coding SNPs (32,388)	Combination (46,585)
Chipping quality	0.527 ± 0.010 (1.027)	0.519 ± 0.010 (1.024)	**0.544 ± 0.011** (**1.052**)	0.536 ± 0.011 (1.046)
Length/width ratio	0.482 ± 0.010 (1.012)	**0.495 ± 0.005** (**1.025**)	0.493 ± 0.009 (1.042)	0.489 ± 0.010 (1.022)
Senescence	**0.347 ± 0.012** (**1.011**)	0.346 ± 0.015 (1.006)	0.315 ± 0.008 (0.967)	0.339 ± 0.008 (0.997)
Dry matter content	0.735 ± 0.004 (1.016)	0.743 ± 0.002 (1.023)	**0.746 ± 0.003** (**1.028**)	0.744 ± 0.003 (1.029)
Yield	**0.280 ± 0.014** (**0.970**)	0.242 ± 0.011 (0.901)	0.239 ± 0.010 (0.885)	0.253 ± 0.013 (0.920)

Highest obtained correlation for each trait in bold. Bias in brackets.

In general, reducing the number of markers used to model GEBVs disintegrated the model performance at low marker counts, with the highest obtained correlation coefficient of the trait being proportional to the number of reductions tolerated before model collapse. Model collapse was indicated by reduced mean correlation coefficients and substantial widening of variance. For traits with robust, high prediction accuracy, like dry matter content, as few as 1,000 markers were able to produce correlation coefficients close to the best obtained performance, while 10,000 markers are required to model chipping quality and length/width ratio to optimum prediction accuracies, closest approximating the trait pedigree narrow sense heritabilities. For senescence and yield, with generally low prediction accuracy models, increasing the number of markers will improve model performance.

Filtering the markers according to annotation did not notably improve or worsen model performance for any of the analyzed traits, in either top performance (with a high number of markers)—except for yield—or model collapse during marker reduction.

The ability to predict the amplitude of the phenotypic variance, prediction bias, was evaluated for each model as the slope (β) of the regression line between the predicted (x) and observed (y) phenotypic values (**Figure 4B**) and was quite robust. For all traits and across all annotation subsets, the biases approximate 1 regardless of the number of markers included, except for<100 markers. However, a slight dip in bias below 1 and subsequent re-stabilization at ~1 were observed for intermediate marker counts for both senescence and yield—increasing axis resolution reveals that the same trend can be observed for all traits (not shown), indicating that the GEBVs became less biased with increasing model fitting using ~10,000 markers. Using as many markers as possible produced the most reliable prediction accuracies, while a reduced set can still yield models of similar performance for some traits.

### 3.5 Genome-wide association studies

**Figure 5:**
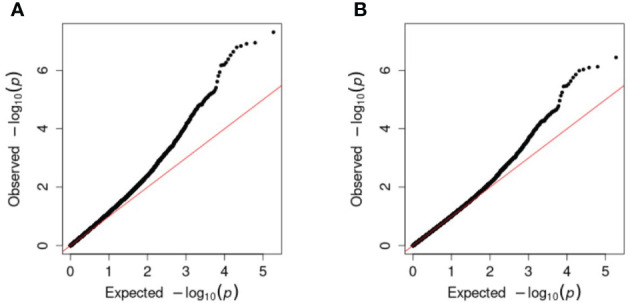
Q–Q plots of expected -log_10_(*p*-values), assuming no marker–trait association, against observed (chipping quality and the full combination set in this example). **(A)** Before correction with the genomic inflation factor and **(B)** after correction.

Single-marker regression GWAS was conducted chromosome-wise for the full combination set of 93,170 SNPs for each trait (**Supplementary File 7**) as well as for non-synonymous, synonymous, non-coding, and a 1-in-3 reduction of the combination set (**Supplementary Figures 6-10**, **Supplementary File 8**). Population structure was accounted for by including **G**-matrices based on all chromosomes, except the one encoding the marker being tested for association. The Q–Q plots of the observed *versus* expected -log_10_(*p*-value) for each analysis showed some inflation of the *p*-values from the expected, assuming no association (**Figure 5A**) for most traits, which was why the genomic inflation factors (*λ_gc_*), ranging from 1.03 to 1.17, were used to correct the *p*-values for traits where *λ_gc_* >1 (**Figure 5B**). Deviation in the tail after correction indicated that significant marker effects were found.

**Figure 6:**
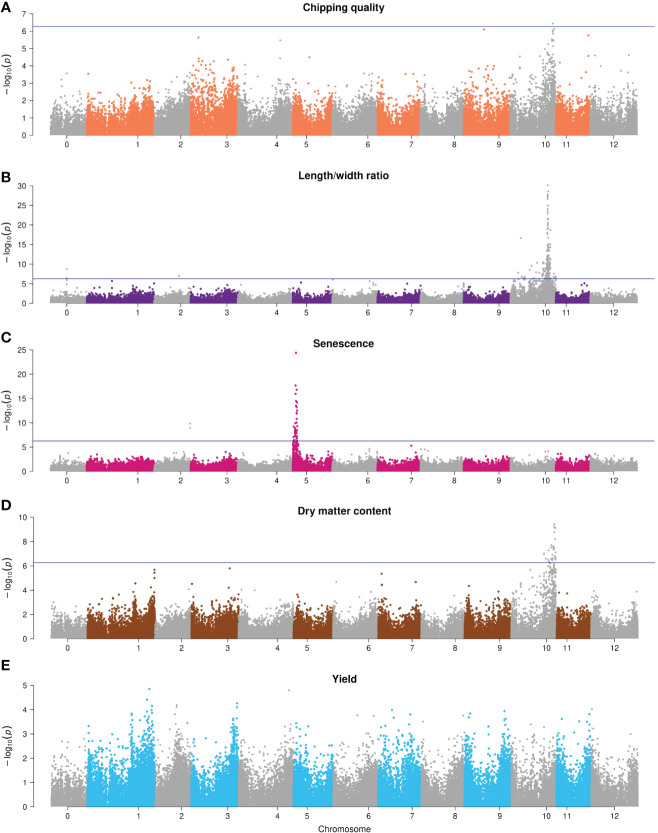
Manhattan plots of -log_10_(*p*-value) GWAS results of the full 93,170 marker annotated combination set. **(A)** Chipping quality, **(B)** length/width ratio, **(C)** senescence, **(D)** dry matter content, and **(E)** yield. Chromosome 0 is pseudomolecules. The significance threshold with Bonferroni correction is indicated with a horizontal line.

The Manhattan plots of the corrected -log_10_(*p*-values) are shown in **Figure 6** for the full combination set. For chipping quality, a single significant marker was identified on chromosome X (*p*-value = 3.6*10^-7^), explaining 3.9% of the total phenotypic variance.

The length/width ratio phenotypes follow ratio distribution, but regardless we have used Bonferroni correction for correction for multiple testing, and the weak QTL peaks for the trait might be false positive associations from lenient correction. Two regions with significant SNPs were found for length/width ratio—a single SNP on chromosome II (*p*-value = 1.1*10^-7^, Chr2;33033331, 3.7% explained variance), while significant associations were detected almost chromosome-wide for chromosome X (142 in total), but most densely around 48 Mb, with a distinct peak of *p*-value = 1.9*10^-30^, explaining 16% of the phenotypic variance. A region of three same-gene SNPs in the unmapped pseudomolecules (chromosome 0) (*p*-value = 2.3*10^-9^, PGSC0003DMG400026855) was also found but has been mapped to chromosome X (48.6 Mb) in the DMv6.1 reference genome (Pham et al., 2020) and is hence part of the 48-Mb major QTL.

Dry matter content also displayed a region of significant SNPs on chromosome X between 48 and 58 Mb (peak *p*-value = 3.7*10^-10^, explaining 5.7% phenotypic variance).

For senescence, two regions of significant SNPs were identified: two same-gene SNPs on chromosome II (*p*-value = 1.4*10^-10^, PGSC0003DMG400012642, 5.5% explained phenotypic variance) and a major peak on chromosome V from 10 to 60 Mb with peak *p*-value = 3.9*10^-25^ that explained 13.7% phenotypic variance. No significant associations were found for yield.

GWAS was also performed for four of the marker sets, namely, the non-synonymous, synonymous, non-coding, and 1-in-3 reduced combination SNP sets, each being ~30,000 SNPs. Generally, all data sets yielded similar results; however, a positional shift of the major QTL could be observed for all traits using the ncSNP sets (with the lowest SNP density in genic regions and highest centromeric SNP density) either from a distal arm position to the pericentromeric region of that chromosome (e.g., for senescence) or to a distal position in another chromosome (e.g., for length/width ratio and dry matter content). In addition, associations on different chromosomes outside the signals could be observed for all traits and were found to differ in position across datasets.

The observed QTLs are in close agreement with those previously observed for the traits analyzed. Chipping quality is dependent on the genetically controlled mechanism of cold-induced sweetening (CIS) that is caused by reducing sugar accumulation in the cold storage of tubers (Fischer et al., 2013; Xiao et al., 2018). Maillard reaction during frying affects the quality of chips and French fries (D’hoop et al., 2014). The reducing sugars glucose and fructose are dissimilated from amyloplastic storage starch granules to serve as osmo- and cryoprotectants as part of the tuber starch metabolism during CIS (Schreiber et al., 2014; Van Harsselaar et al., 2017), which explains why chipping quality and starch content are correlated traits. Studies have identified loci on all chromosomes associated with frying color, and indeed several of the genes affecting the trait are related to starch and sucrose metabolism (Li et al., 2008; Werij et al., 2012; Fischer et al., 2013; D’hoop et al., 2014; Xiao et al., 2018; Byrne et al., 2020). Using nsSNPs, a SNP on chromosome III at 38.4 Mb was associated with chipping quality, located proximal to the *Pain-1* invertase (PGSC0003DMG400013856, 39255023-9538), involved in enzymatic sucrose conversion into reducing sugars (Draffehn et al., 2012), which has previously been associated with chip quality and tuber starch content (Li et al., 2008; Draffehn et al., 2010; Schreiber et al., 2014). Chipping quality-associated SNPs on chromosome X at 55.4 Mb (combination set) and 58–59 Mb (nsSNP, sSNP) were located within the starch metabolism gene-rich region at 50–60 Mb (Sharma et al., 2013). Previous studies have also found fry color and starch content associations in this region of cell wall invertases (e.g., in the *Inv-ap-a* locus of *InvCD111* and *InvCD141* at 55.8 MB), invertase inhibitors (e.g., *InvInh-10/4*), a sucrose phosphatase, a fructose-1,6-biphosphatase, a fructose-biphosphate aldolase, and patatins (the main tuber storage proteins) (Li et al., 2008; Draffehn et al., 2010; Schreiber et al., 2014; Byrne et al., 2020). We found numerous associations for dry matter content in this region, consistent with the traits being correlated through the shared metabolic pathway. Additionally, single-SNP chipping quality associations on chromosome XI at 42.7 Mb (1-in-3 combination) and chromosome XII at 1.5 Mb (nsSNP) were proximal to genes functional in plant starch interconversion (Schreiber et al., 2014), namely, invertase *Inv-n-11/3* (PGSC0003DMG400026530, 39907597-13829) and *AGPaseB-12* (PGSC0003DMG400046891, 1226599-30218), respectively. A selection of single SNP associations for dry matter content could also be found in using the different annotation data sets. On chromosome III, a dry matter content association was found (50.3 Mb, 1-in-3 combination) downstream of the *SEX4* phosphoglucan phosphatase (PGSC0003DMG400015246, 50875724-85587)—a region at 50.8 Mb where Wilson et al. (2021) have also found associations to dry matter content. The nsSNPs and ncSNPs gave significant associations on the north arm of chromosome XI (0.5–4 Mb and 7.1 Mb). For the ncSNPs alone, no association was seen on chromosome X, indicating missing linkage on that chromosome when using only ncSNPs. The upstream region of chromosome XI is scattered with starch and sucrose conversion genes, e.g., *UGPase-11* (PGSC0003DMG401013333, 808268-14810), sucrose transporter *Sut1* (PGSC3000DMG400009213, 9052433-7333), invertase inhibitors *InvInh-11* (PGSC0003DMG400038811, 7433845-4381), invertases *INV-11/1* (PGSC0003DMG400019494, 5046813-52945), and debranching enzyme *DBE-11* (start 3945240, not annotated). *UGPase-11* was screened by Schreiber et al. (2014), who did not find an association in a 208-genotype tetraploid population. While not reproducible in more than two of the annotated data sets, the associations on chromosome XI do coincide with genes involved in trait-related processes.

Length/width ratio has a major QTL peak at ~48 Mb on chromosome X. The major QTL controlling overall tuber shape was identified as the *R_0_* locus (Van Eck et al., 1994), and trait effect has been mapped to this region in previous studies (Endelman and Jansky, 2016; Manrique-Carpintero et al., 2018; Zia et al., 2020). Most recently, the *R_0_* locus was fine-mapped to a 200-kb region from 49.5 to 49.7 Mb in the DMv6.1 reference genome (Pham et al., 2020) spanning 18 candidate genes. RNA sequencing indicated that five genes had differential gene expression, including genes of a lipid transfer protein and a HSI2-like protein, both with roles in plant growth hormone response, regulating plant growth and development (Fan et al., 2022). It is uncertain whether the remaining associations for length/width ratio on chromosome X are spurious hits because of linkage to the *R_0_* locus or true associations without additional fine-mapping. Using the ncSNPs alone reveals a major QTL on chromosome XI from 0.1-4.1 Mb and 9.6 Mb rather than on X. Tuber shape QTLs have, however, previously been identified on the north arm of chromosome XI (D’hoop et al., 2014; Manrique-Carpintero et al., 2018), which is why it cannot be refuted as a true positive data-dependent association. Additional tuber shape associations were found using the four SNP sets on chromosomes I, II, VI, and VII. While D’hoop et al. (2014) found tuber shape associations in the upstream region of chromosome II, the downstream end of chromosome VI (Manrique-Carpintero et al., 2018), and Park et al. (2021) identified a QTL on chromosome VII, the associations found in chromosome I (sSNP) appear previously undetected.

Senescence is a major QTL effect trait. Deletion alleles in the *StCDF1* gene (PGSC3000DMG400018408, 4538880-41736) in the north end of chromosome V result in early maturing plants. The gene encodes a transcription factor, mediating between the circadian clock and the tuberization signal (Kloosterman et al., 2013). We identified this major QTL, with a total of four within-gene SNPs having a significant association in the four analyses. Additional associations were also found on chromosomes II, III, and VII using different SNP sets. A maturity trait association has previously been found on chromosome II (D’hoop et al., 2014), but not on III or VII. Interestingly, the associations found between 2.8 and 2.9 Mb on chromosome III (nsSNPs) are located upstream of *auxin response factor 8-1* (PGSC3000DMG401018664, 2955745-64135) and two auxin hydrogen symporters (PGSC3000DMG400018678, 2976443-2982482 and PGSC3000DMG400018668, 3084832-91586). In addition to short-day photoperiods, tuberization and plant development are under hormonal control, and high yield is correlated with late plant maturity (van Eck, 2007). Auxin affects growth, the rate of tuberization, and cell differentiation at all stages of life, but plant response is genotype dependent (Kolachevskaya et al., 2019). A confirmation of these candidate genes as senescence QTLs requires further analysis.

## 4 Discussion

### 4.1 Marker type and density consequences for genomic prediction

We found that the performance of GS was generally insensitive to marker types. It was somewhat surprising that the ncSNPs expected to have the least importance for phenotypic expression were equally capable of prediction performance as nsSNPs, which have a more direct relationship with phenotypic performance, given that each nsSNP directly causes an amino acid change in the gene product. However, other studies on the impact on genomic prediction of using rare and low-frequency variants in dairy cattle (Zhang et al., 2018) and functionally prioritized variants in maize (Ramstein and Buckler, 2022) also reported a lack of model improvement. This indicates that there is sufficient marker density to adequately describe the relevant genomic variation independently of how markers are filtered. In fact, the distance of the marker to causal polymorphisms, as well as the density of markers needed to characterize the population, is related to the LD span in the genome (Abera Desta and Ortiz, 2014) rather than to the much higher number of individual polymorphisms. Vos et al. (2017) estimated the LD block size to be 0.6–1.5 Mb and even up to 2.5 Mb in introgressed regions in an analysis of 537 tetraploid cultivars. Coarsely extrapolating these results to this study and assuming an average LD block size of 1 Mb in the MASPOT population, this would suggest that SNPs within a window of 500 kb on either side of a causal polymorphism are reliable as markers. Such a large window size includes multiple markers of any of the types analyzed and suggests that performance difference between marker types can only be expected when comparing more distantly related genotypes, where the LD block size is smaller. In summary, the linkage between the causal alleles and neutral variants is likely sufficiently strong to render the effect of concentrating the causal variants mute, with the causal variant effects becoming diluted in their linkage patterns.

It was also found that the minimum number of markers required to satiate the model was dependent on the trait considered. Traits with lower maximum prediction accuracies, e.g., yield and senescence, still showed improvement in prediction performance when using SNP densities higher than 10k markers as compared to traits of intermediate-high heritability, where training on 10k markers amply reached model optimum performance. Indeed for the high-heritability trait dry matter content only ~1k SNPs were needed to border the plateau optimum. Yield also represents an exception to the rule that marker type is unimportant. Using nsSNPs compared to the full combination set leads to a 12% improvement of model performance at 30k marker density and 16% compared to the worst-performing ncSNPs. Furthermore, the nsSNP model for yield, in contrast to all other traits, was consistently more resilient to marker reduction than models based on other marker types. We speculate that the reason for this difference in behavior is that yield is genetically the most complex of the traits and that the developed models are, in fact, only metastable, the nsSNP model being the most stable and thus most resilient to marker reduction. An alternative strategy to filtrating markers by type alone could be identification of candidate causal variants (Zhang et al., 2018) by, e.g., bioinformatics analysis (Lee et al., 2020; Høie et al., 2022) based on homology and/or predicted structural information to drive filtration and introduce weighting according to predicted functional impact.

### 4.2 Dry matter content heritability estimate discrepancy

Notably, we observed a substantial difference between genomic and pedigree heritability estimates for dry matter content, a trait known to be highly heritable (Ortiz et al., 2023). This indicates that some genetic variance is not captured in the **G**-matrix-based model employed in GS. This might stem from cryptic data structures resulting from phenotypic grouping, e.g., according to starch content performance, during breeding and subsequent in-group crossing, generating a systematic SNP × group interaction that is not captured in the heritability estimation, where SNP effects on phenotype are differential across groups, and the average SNP effect across the panel does not capture the variance. However, the specific reason for the observed genomic heritability deflation remains obscure. As a consequence, we only evaluate the performance on genomic prediction models relative to the pedigree narrow sense heritabilities.

### 4.3 Pedigree heritability *versus* genomic prediction

Correlations of 0.54 and 0.75 were reproduced for chipping quality and dry matter content, respectively, from those reported for GBLUP models trained on the MASPOT panel in Sverrisdóttir et al. (2017, 2018). These results are also consistent with those found in other studies using different statistical models (Stich and Van Inghelandt, 2018; Byrne et al., 2020; Wilson et al., 2021; Ortiz et al., 2022; Pandey et al., 2023). However, from the pedigree narrow-sense heritabilities, higher correlations of up to relations of 0.86 and 0.95 could be observed for chipping quality and dry matter content, respectively. Similarly, the highest obtained correlation for length/width ratio of 0.49 was somewhat lower than the theoretical maximum attainable of 0.77 based on pedigree narrow-sense heritability. A rather poor model performance with correlation of 0.35 was also obtained for senescence using GBLUP, as could be expected for a single-gene trait, despite an estimated pedigree narrow-sense heritability of 61% for the trait, i.e., a theoretical maximum correlation of 0.78. In summary, this indicates that some additional additive variations are still not captured by the prediction models. For the single, large effect QTL traits length/width ratio (Fan et al., 2022) and senescence (Kloosterman et al., 2013), including the major QTL associations as fixed effects in the models, might improve prediction (Kim et al., 2022).

At best, yield correlation coefficients were low or modest, falling between 0.24 and 0.28 using GBLUP. The narrow-sense pedigree heritability indicates that 29% of yield phenotypic variance can be explained by additive genetic effects, corresponding to a maximum correlation of 0.55. The relatively poor model performance is likely attributable to the putative metastability of the model. Increasing the number of individuals in the training population is likely needed to improve this in the future. This will presumably reduce the linkage block size and hence increase the resolution of marker–phenotype relationship. Reduced linkage block sizes will, in turn, change the minimum marker density requirements to create stable models compared to those found here.

### 4.4 Marker type importance in GWAS

In contrast to genomic prediction, marker type was important for GWAS analysis. We observed both intra- and inter-chromosomal displacements of major and minor QTL signals when using ncSNPs compared to other marker types. For both dry matter content and length/width ratio, the major QTL signal is transferred from chromosome X to XI. However, the signal amplitude is not diminished, which suggests that a portion of the non-coding part of chromosome X is erroneously mapped to chromosome XI, still allowing full capture of the association signal but translocating it. We speculate that the risk of such errors in mapping to the genome reference model is more likely to happen for non-coding regions than for coding regions due to an enrichment of repetitive elements in intergenic regions (Mehra et al., 2015). Similarly, the inter-chromosomal shift of the chromosome V senescence signal of senescence using ncSNPs could also be a result of mis-mapped sequencing reads. Minor QTL shifts were observed across all marker types, also indicating that the finer resolution of GWAS is indeed marker type dependent. Whether the minor QTL hits produced with different SNP types are true associations or spurious hits, however, is pending verification.

### 4.5 Consequences for breeding

The MASPOT panel clones used to train GBLUP genomic prediction models in this study were not selected for any agronomical performance traits following the full-diallel cross from 18 parents of elite cultivars and breeding clones, representing a diverse selection of alleles from the gene pool and contributing to a broad phenotype range for training. For application in breeding schemes, it is attractive to generate models with high reliability in a broad phenotype range to facilitate both selection and deselection of progeny during breeding selection cycles (Sverrisdóttir et al., 2018). The unbiased correlations obtained for all traits indicate that the range of predicted phenotypic variation is not inflated or deflated compared to the observed, the high-accuracy genomic prediction models being suitable in evaluating both high- and low-performing clones. Using a panel of unselected clones also reduces the potential load of fixed trait-associated alleles in the population, as can be seen in populations of elite breeding material (Kristensen et al., 2018). This can be expected to improve the detection of genome-wide trait associations. However, using a diallel cross as genotype panel also introduces family structure, as is seen in the family-clustered heat map of the genomic relationship matrix (**Supplementary Figure 3**), where clusters of strong genetic relationship between full and half sibs are found along the diagonal. However, the panel is highly heterogenous across families (Sverrisdóttir et al., 2017), and while some sibling-based subgroupings are seen in the PCA plot of the genomic relationship matrix (**Figure 3**), most overlap to form a cohesive group, where the genetic diversity between alleles dominates origin. Regardless, working with populations with family structure, which is almost always the case during potato breeding, necessitates extra caution when evaluating results from association analyses to control false positives. In this study, we have used adjustment by genomic inflation factor in combination with the genomic relationship matrix to successfully obtain robust identification of QTLs.

Based on the evaluated effect on GBLUP prediction model performance imposed by marker density and marker type, respectively, we have found that using 1–10k markers, depending on trait (and possibly population size), distributed evenly across the potato genome, but with no particular demand to location relative to genomic features, is sufficient for the prediction of single traits in tetraploid cultivars. Increasing marker density beyond this level did not notably improve performance gains, particularly for high-heritability traits. This conveniently converges with the marker densities of available SNP arrays like the 20-k SolSTW array (Vos et al., 2015) or the commercial Infinium 12K V2 Potato Array. SNP arrays can generate highly robust genotypes compared to low-depth GBS (Gentzbittel et al., 2019), with ample marker density for high-performance prediction modeling of even low heritability traits, even though for some very complex traits such as yield, large training populations are likely necessary.

In this study, we used a single-trait standard additive GBLUP model for evaluating the impact of marker type and marker density on prediction performance since using a simple model on the individual traits facilitated the interpretation of these effects. However, in breeding programs, clones are selected on multiple traits concurrently, and in such a case, multi-trait models are more suitable to use for optimizing genomic prediction models to support selection (Ortiz et al., 2023).

## 5 Conclusions

The aim of this study was to study the effects of marker type and density on genomic predictions and GWAS of key tuber performance traits and to elucidate prediction accuracy dependency on marker density. Overall, it was found that relatively few markers, 1k–10k, were sufficient to support genomic prediction models in tetraploid potato. This is consistent with most high-throughput marker technologies. Marker type was found to be largely unimportant for genomic prediction but could influence QTLs’ placement in GWAS, where ncSNPs alone do not perform satisfactorily.

## Data availability statement

The data presented in the study are deposited in the Zenodo repository “GBS and phenotype data for MASPOT population, a panel of tetraploid potato clones”, accession number https://zenodo.org/doi/10.5281/zenodo.10143576.

## Author contributions

TA: Conceptualization, Formal analysis, Investigation, Methodology, Project administration, Software, Visualization, Writing – original draft, Writing – review & editing, Data curation, Validation. ES: Conceptualization, Data curation, Formal analysis, Investigation, Methodology, Writing – review & editing, Software, Validation. HK: Data curation, Writing – review & editing, Investigation, Methodology. KN: Conceptualization, Funding acquisition, Methodology, Project administration, Resources, Supervision, Writing – review & editing, Data curation.
