# Supplementary material for: The effect of marker types and density on genomic prediction and GWAS of key performance traits in tetraploid potato
Source: Front Plant Sci. 2024 Mar 8;15:1340189. doi: 10.3389/fpls.2024.1340189 (PMC10957621; doi:10.3389/fpls.2024.1340189)

Supplementary Material

# Supplementary File 5: Marker density histograms in all marker reduction iterations

Histograms depicting the marker density in 1 Mb windows along the 12 chromosomes are shown for one marker reduction bin for each iteration of the marker reduction strategy for each marker type. A density histogram is not shown for the final iteration, following 1-in-10 reduction of a ~150 marker set, as only ~15 SNPs remained in total, roughly corresponding to 1-2 SNPs per chromosome.

## Combination set

### Iteration 0 (complete set)


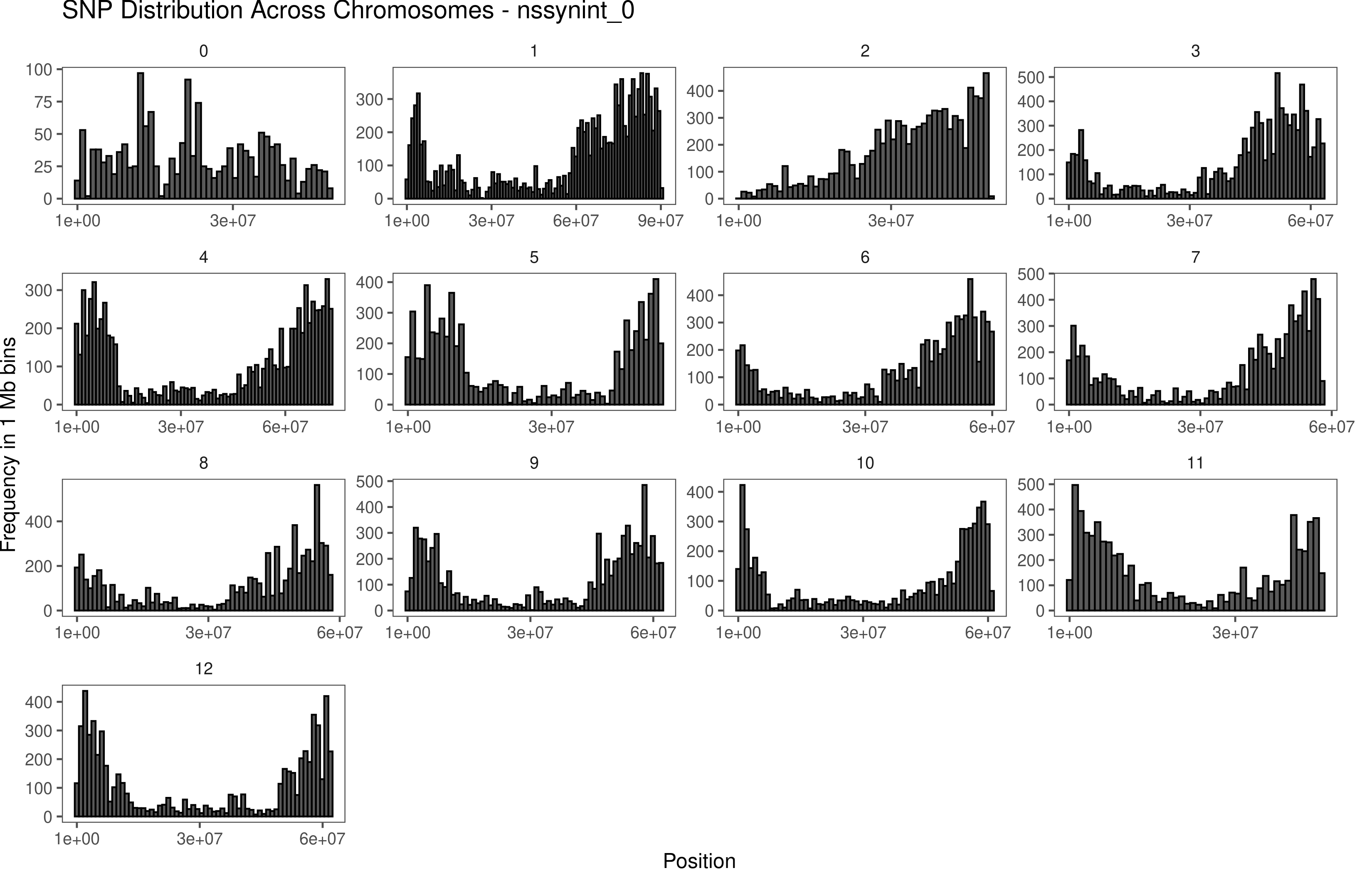


### Iteration 1


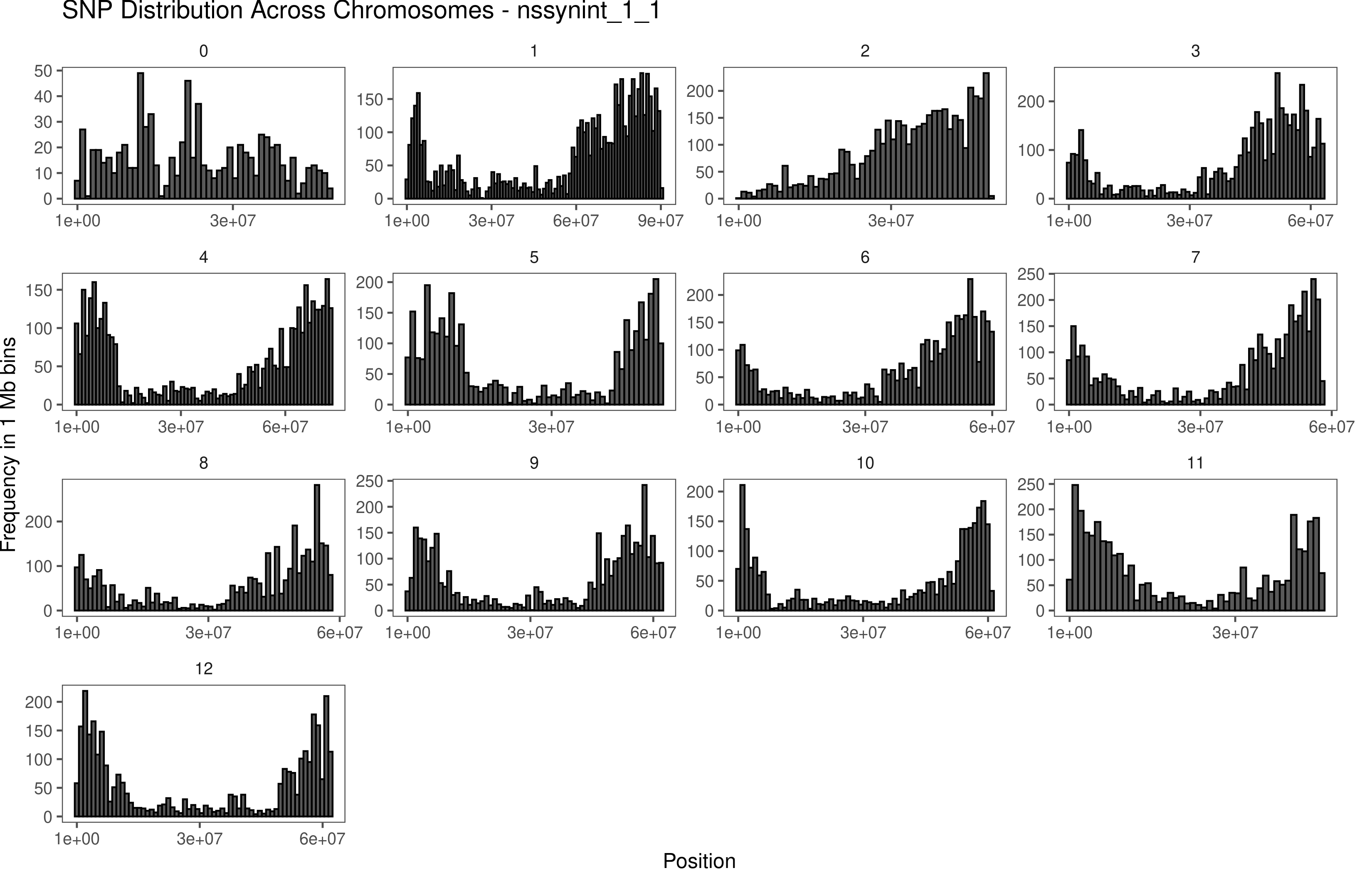


### Iteration 2


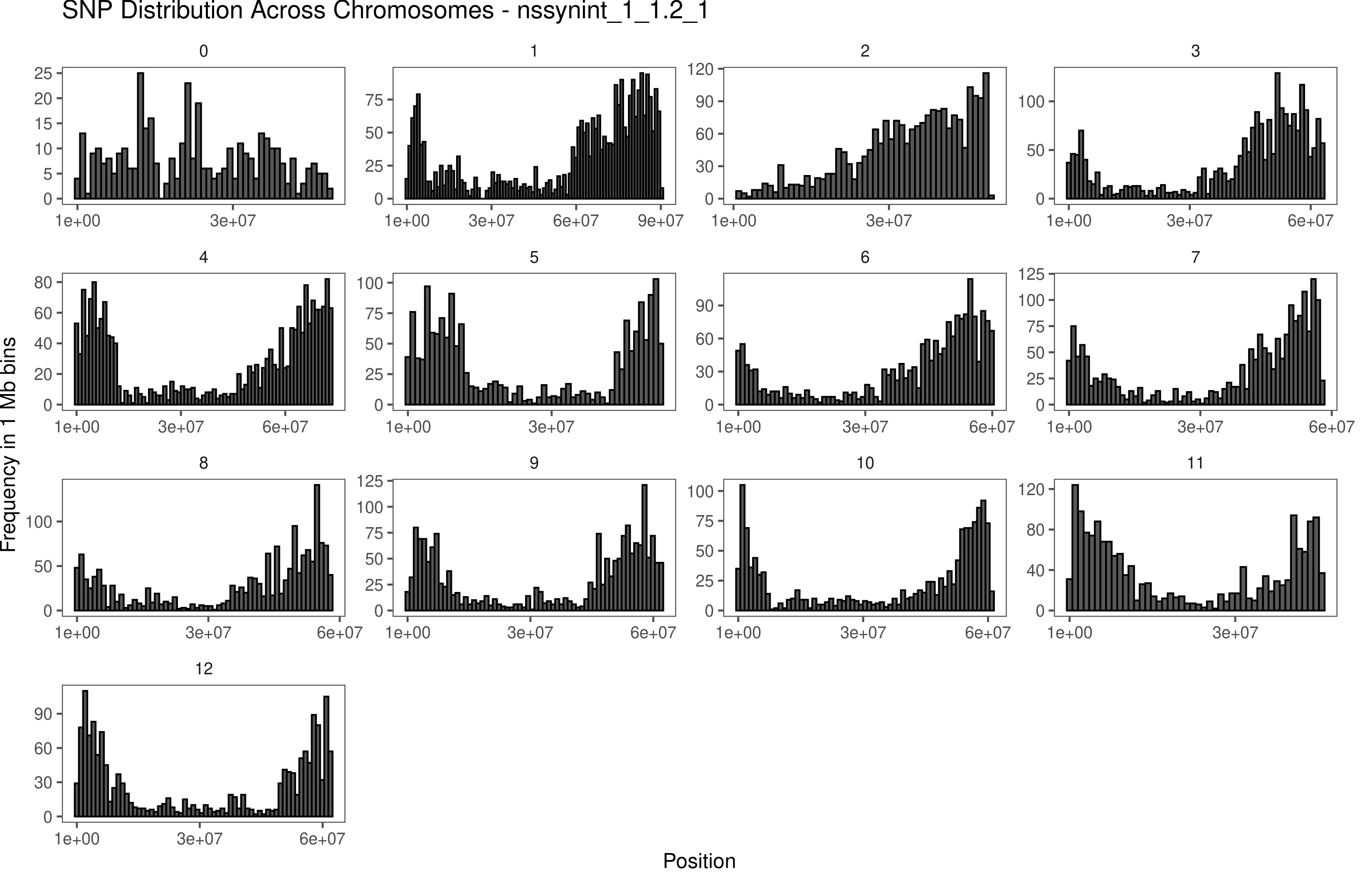


### Iteration 3


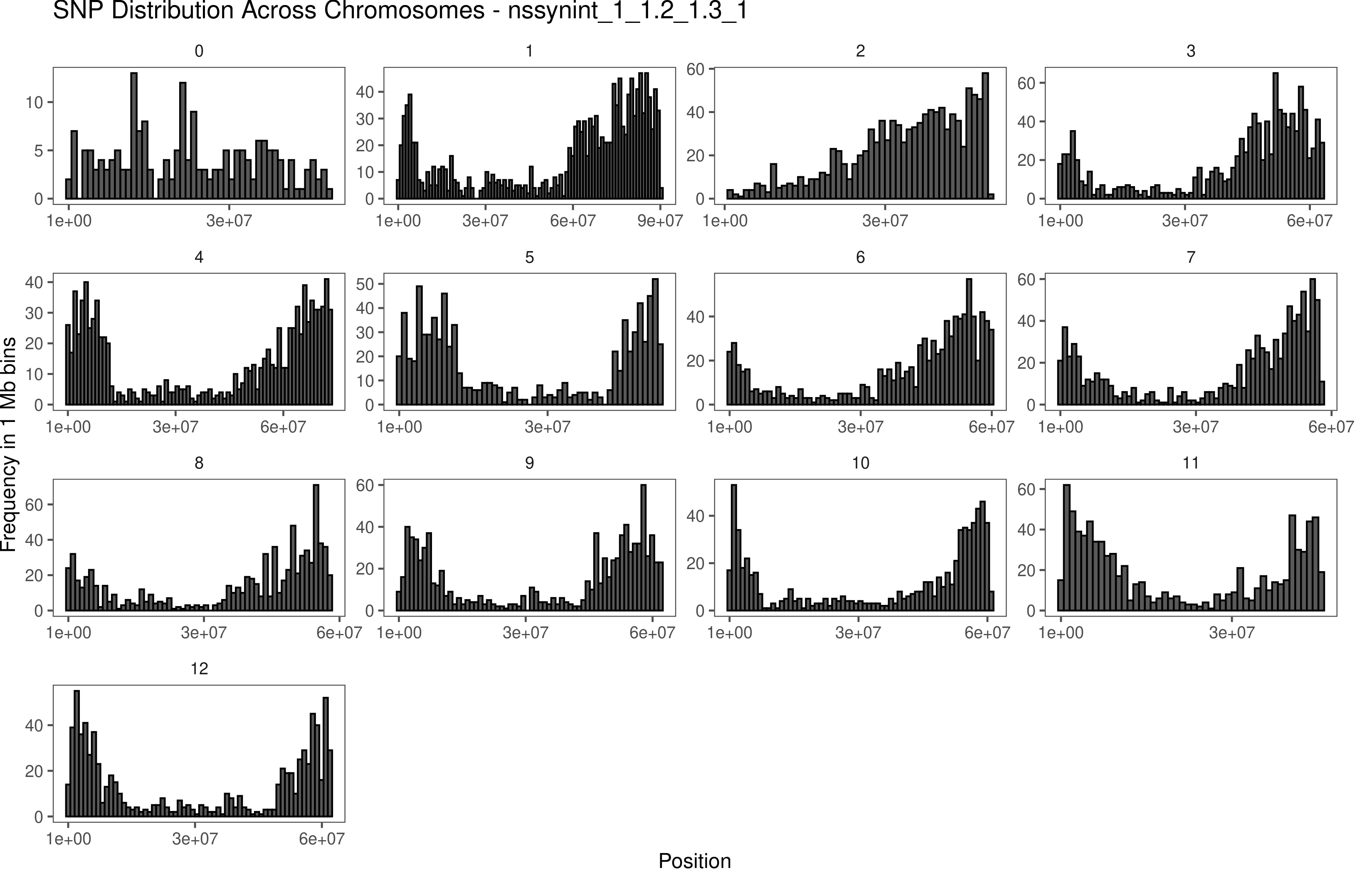


### Iteration 4


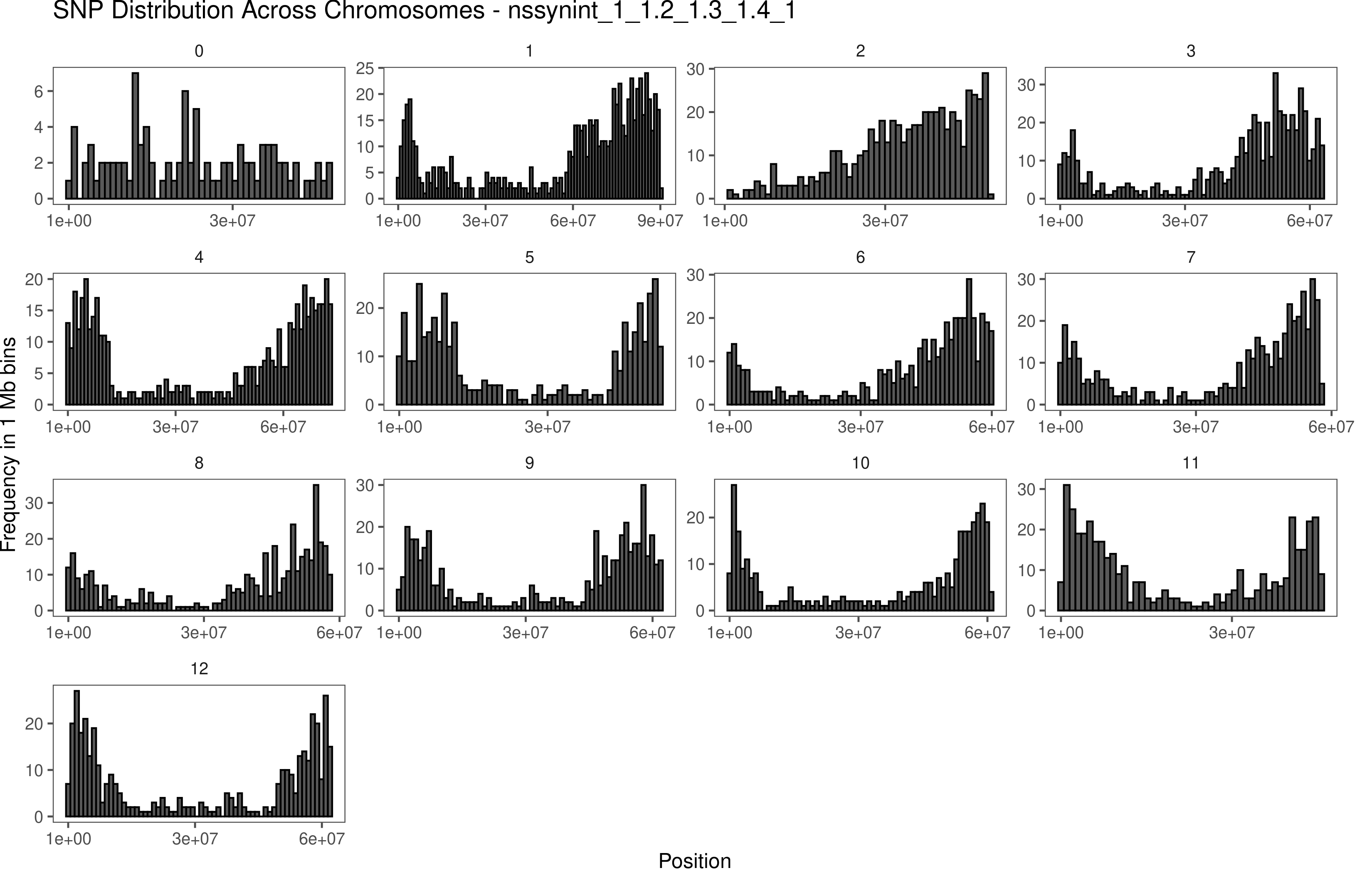


### Iteration 5


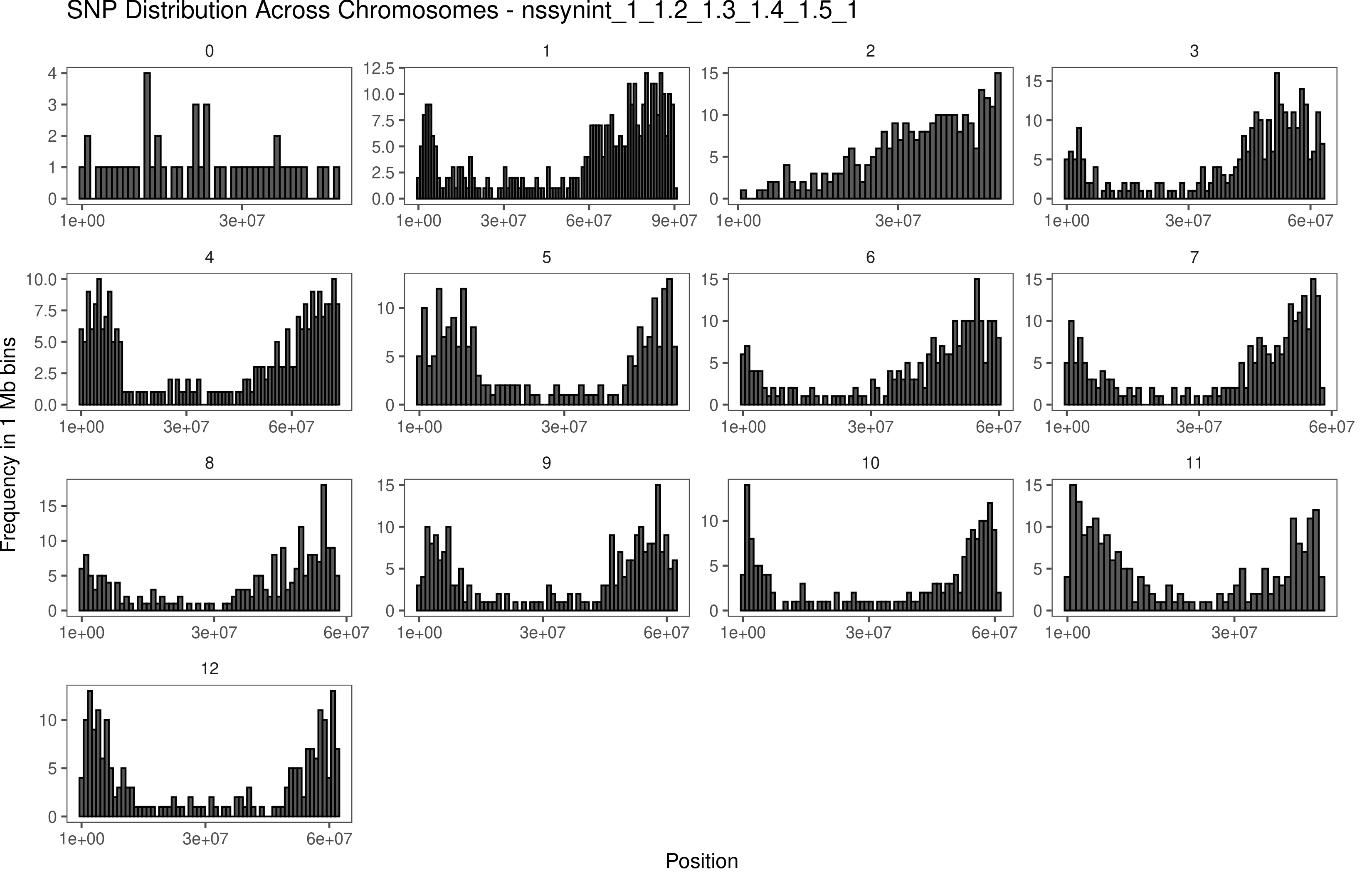


### Iteration 6


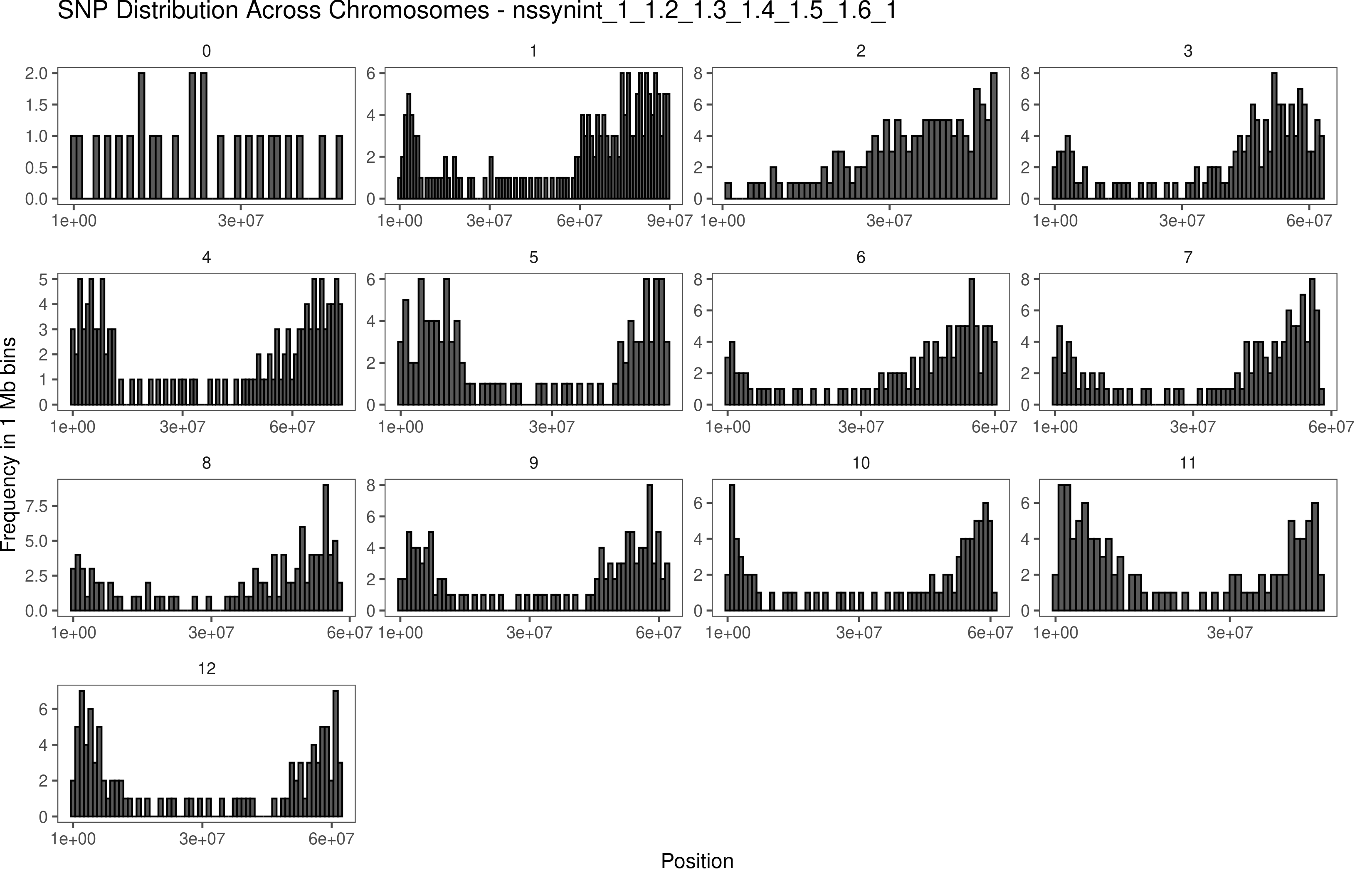


### Iteration 7


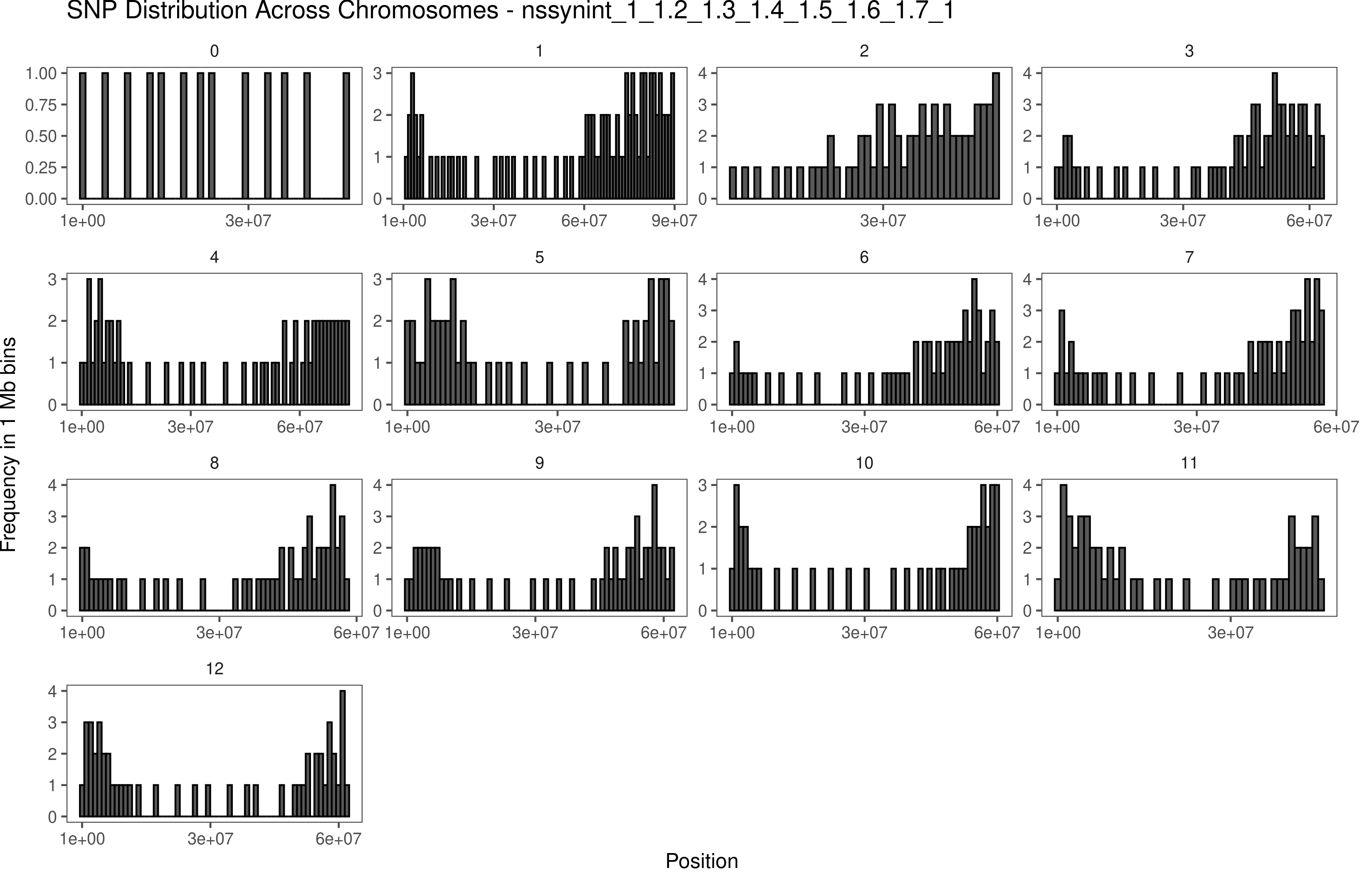


### Iteration 8


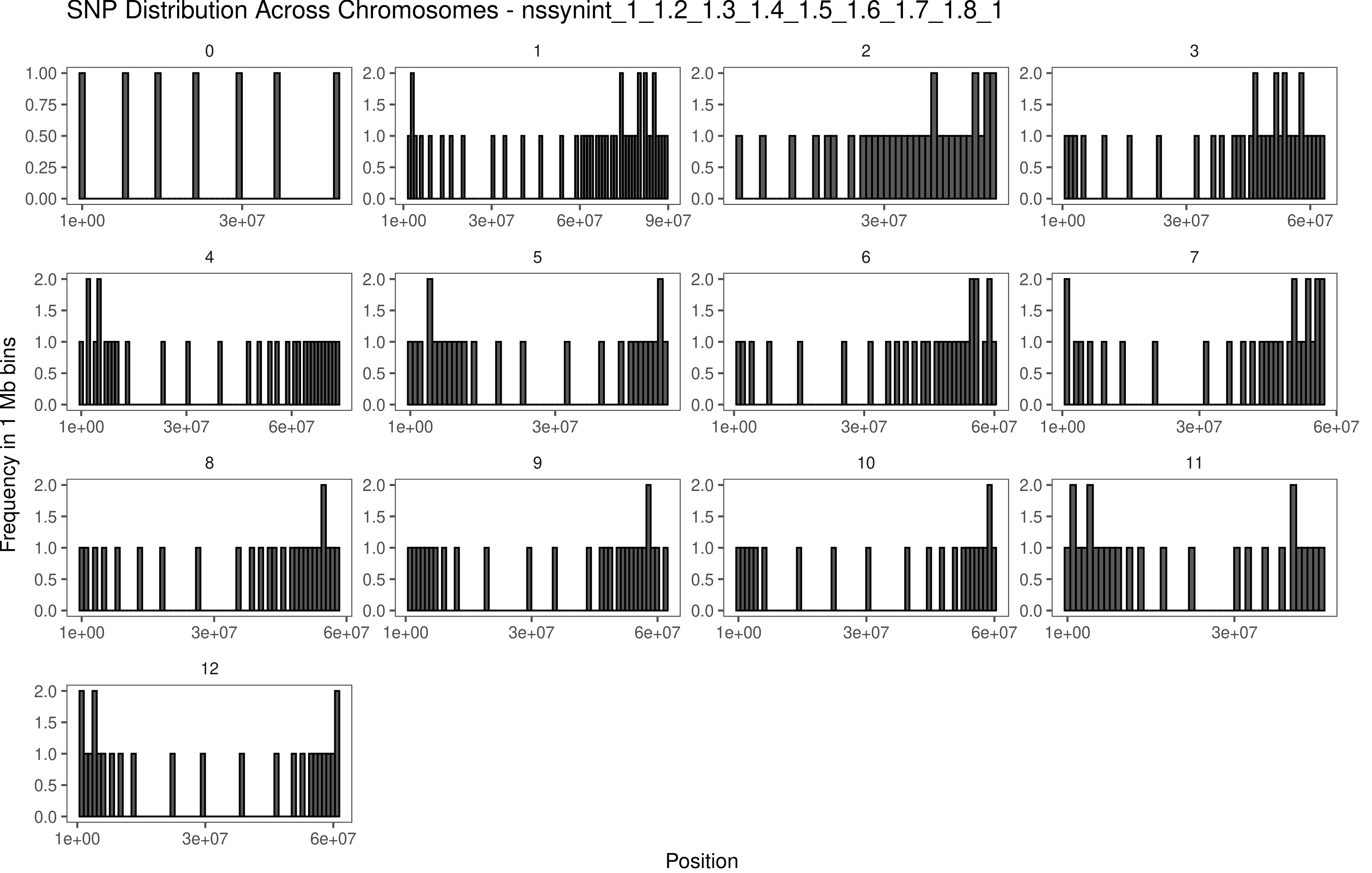


### Iteration 9


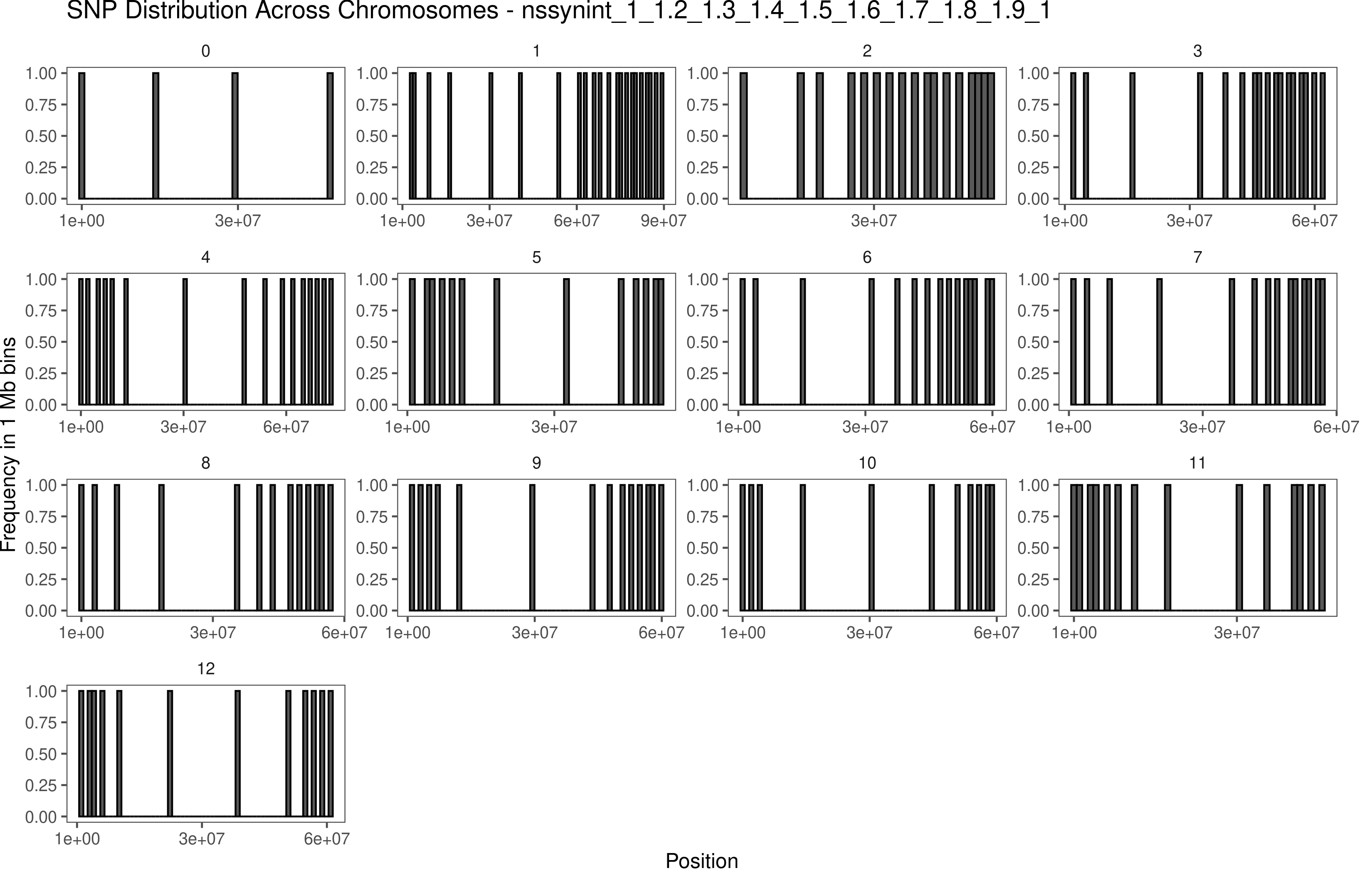


## Non-synonymous set

### Iteration 0 (complete set)


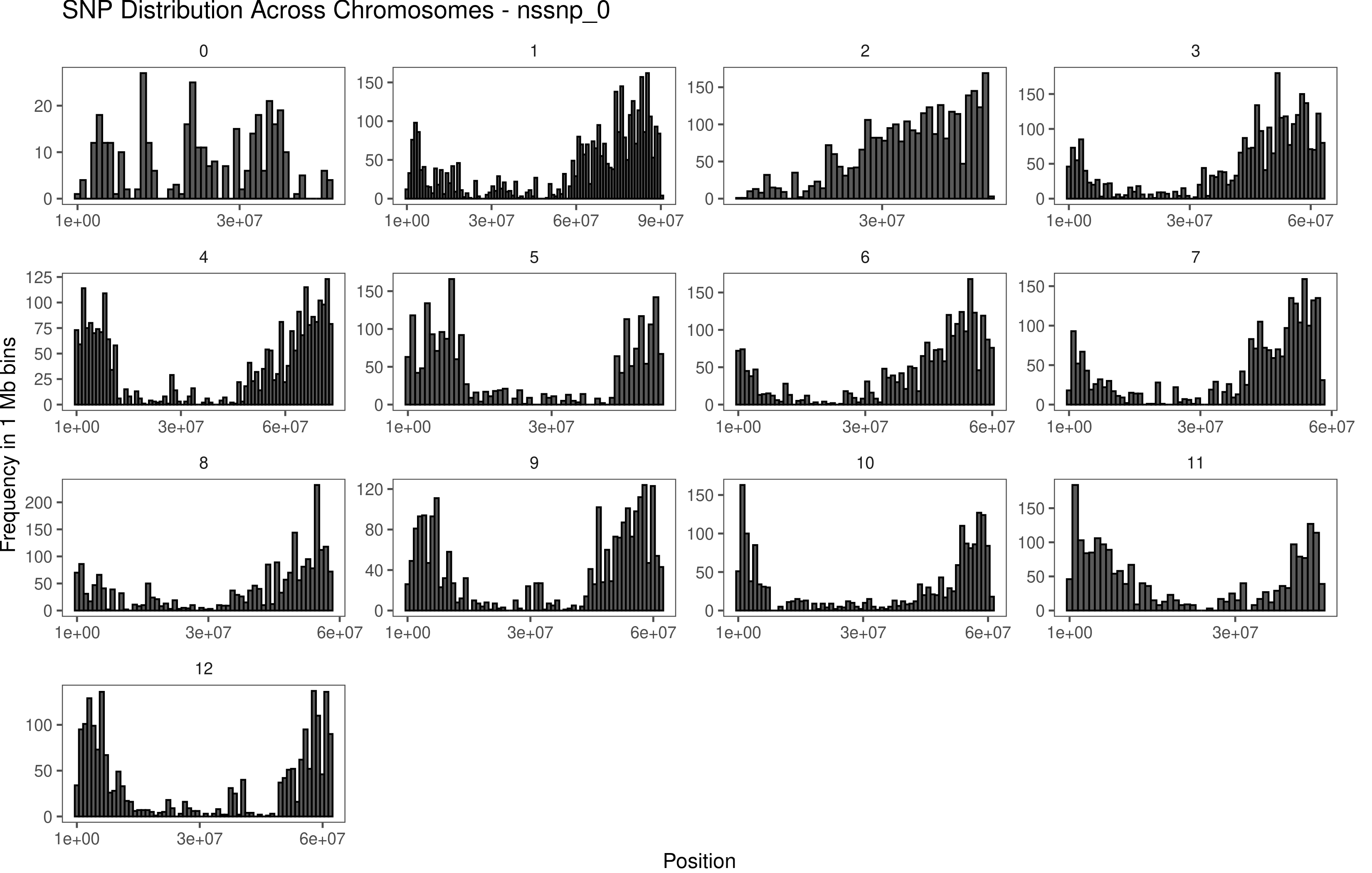


### Iteration 1


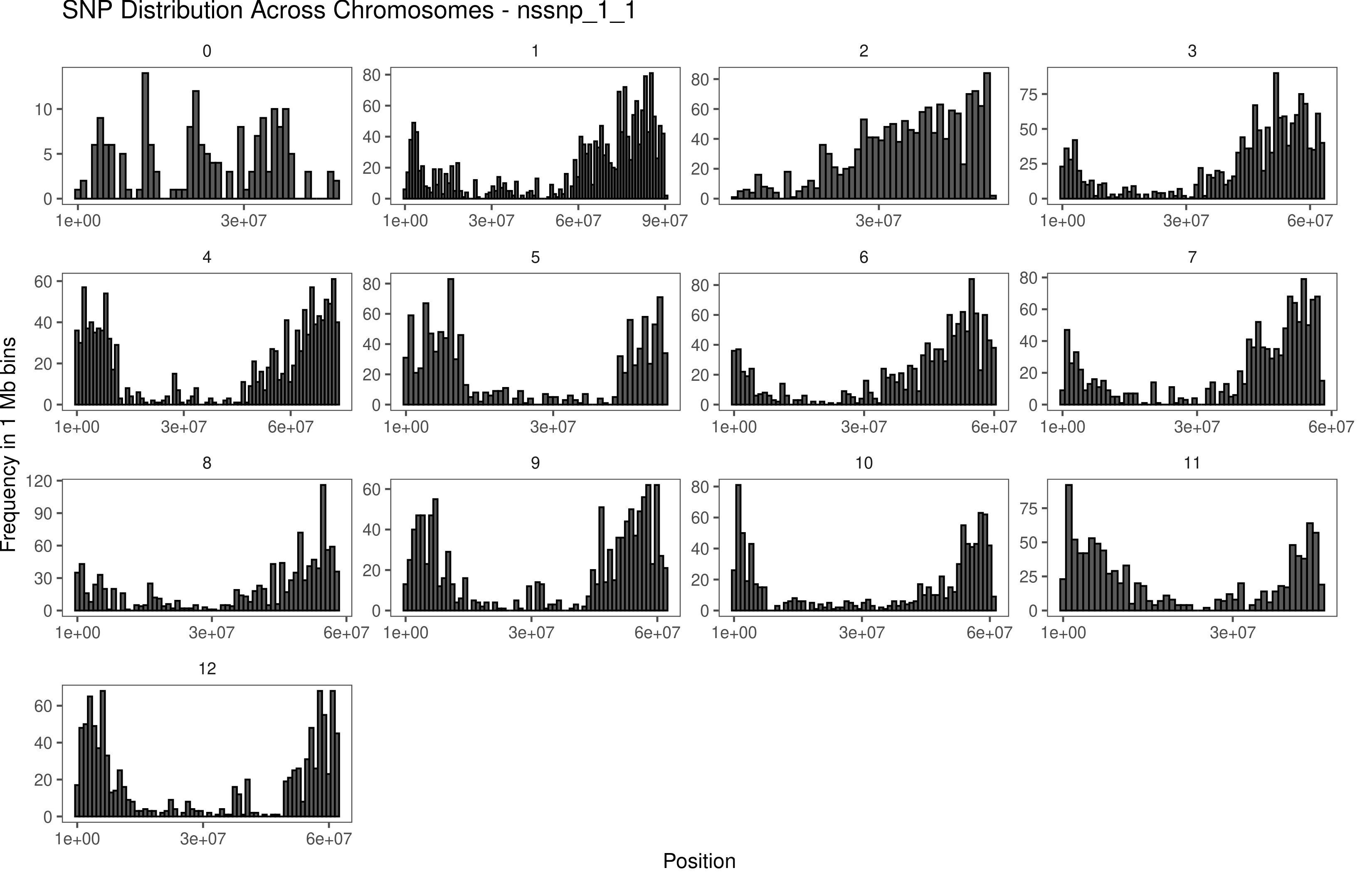


### Iteration 2


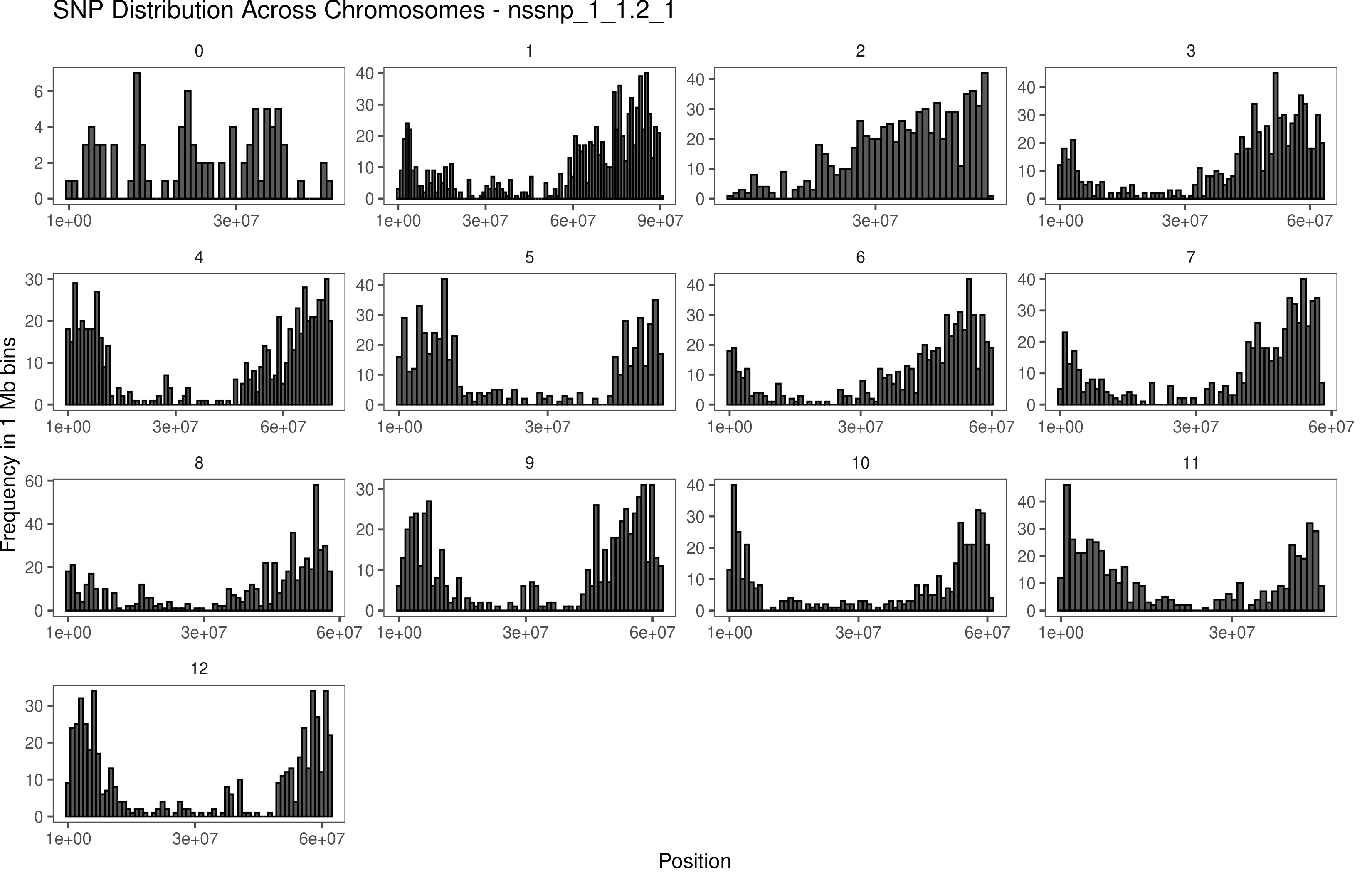


### Iteration 3


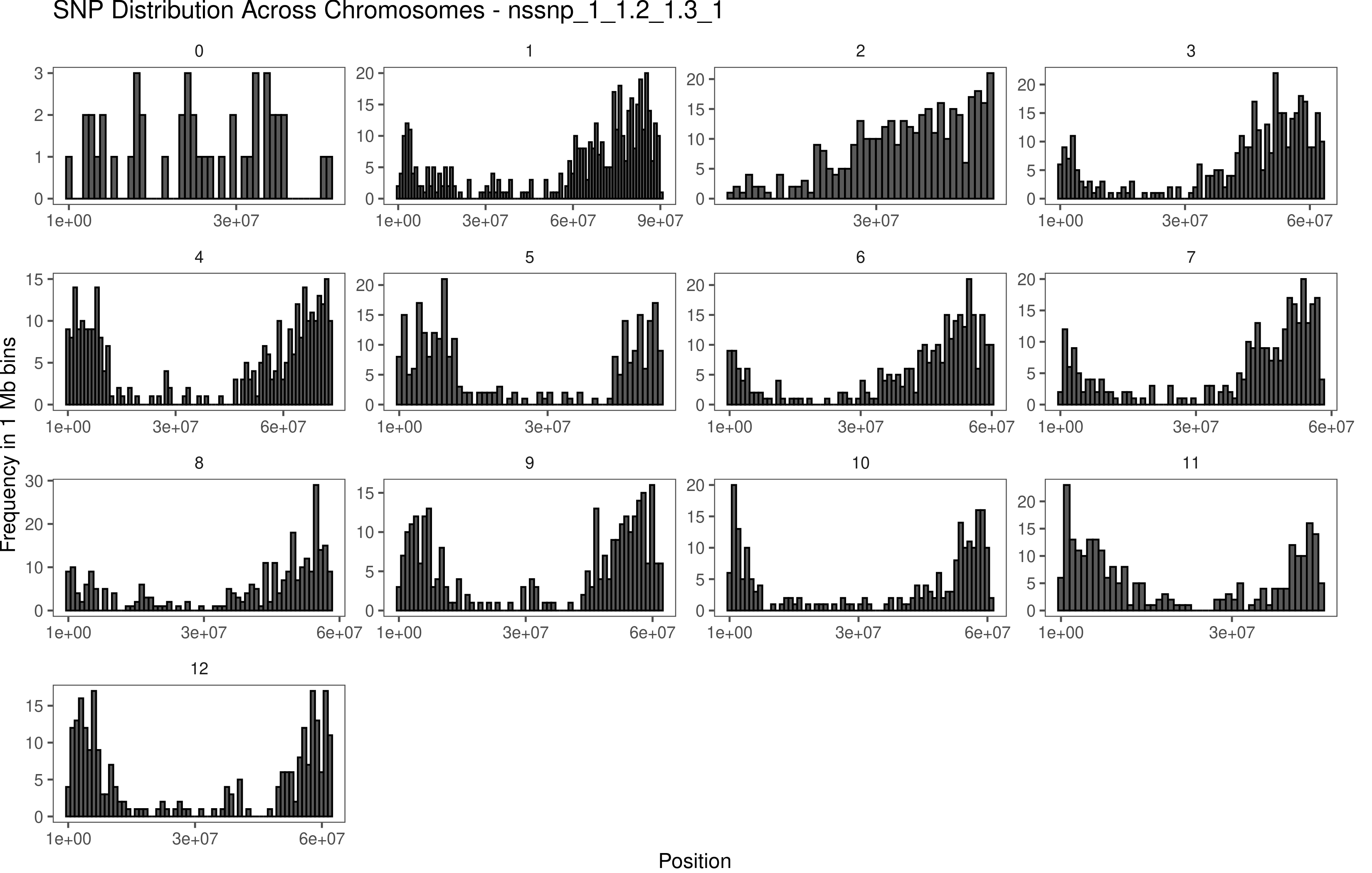


### Iteration 4


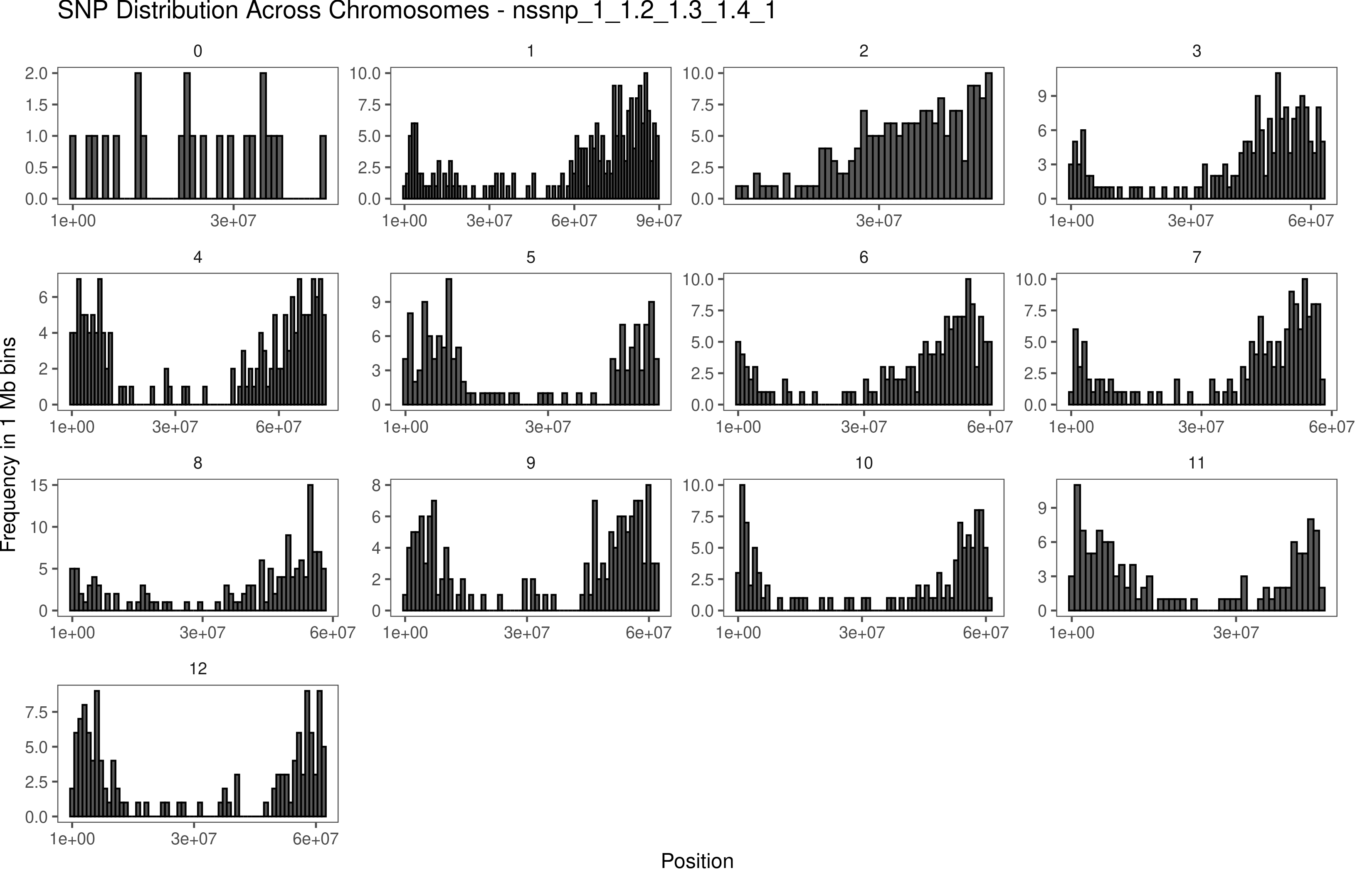


### Iteration 5


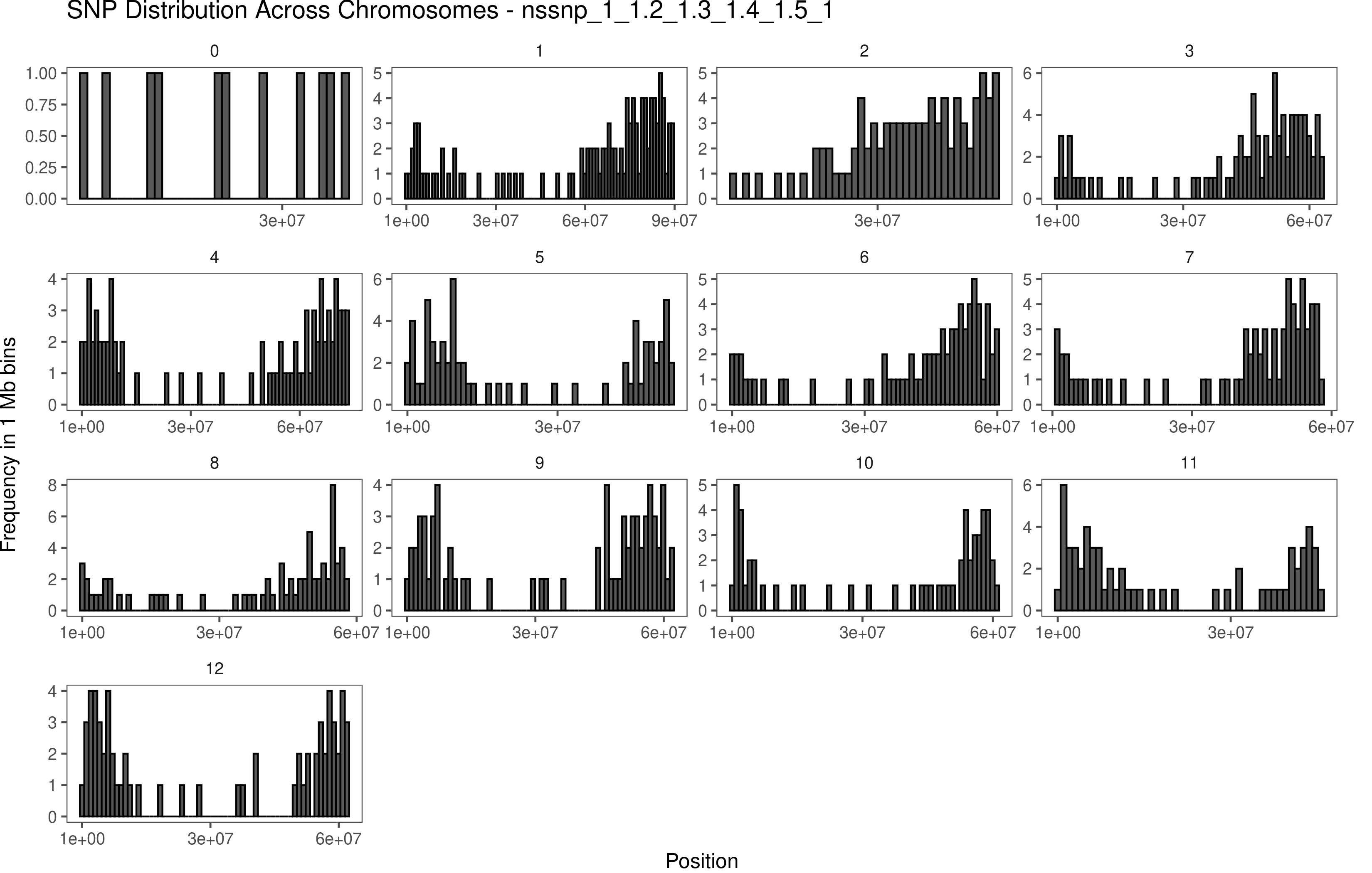


### Iteration 6


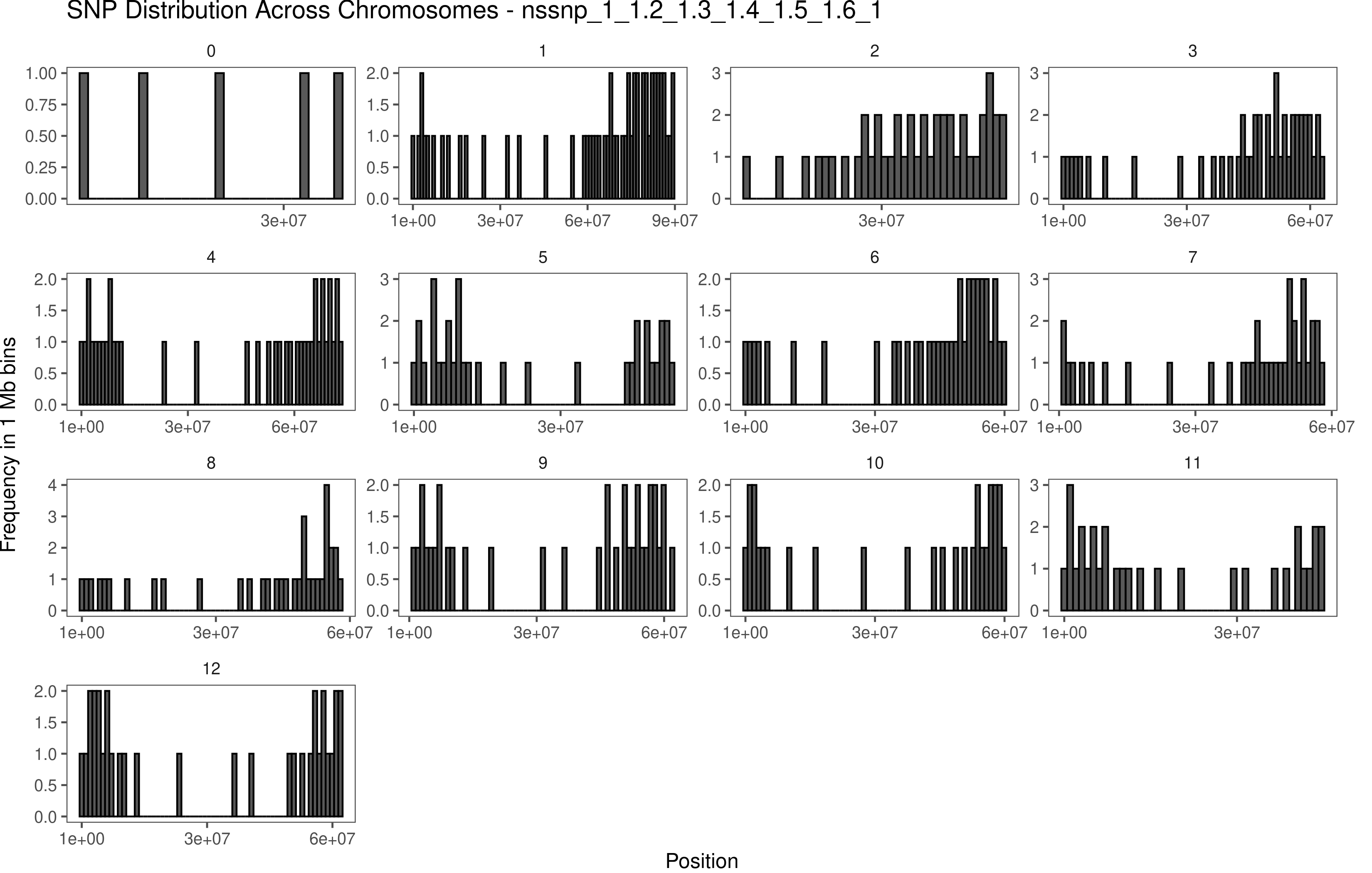


### Iteration 7


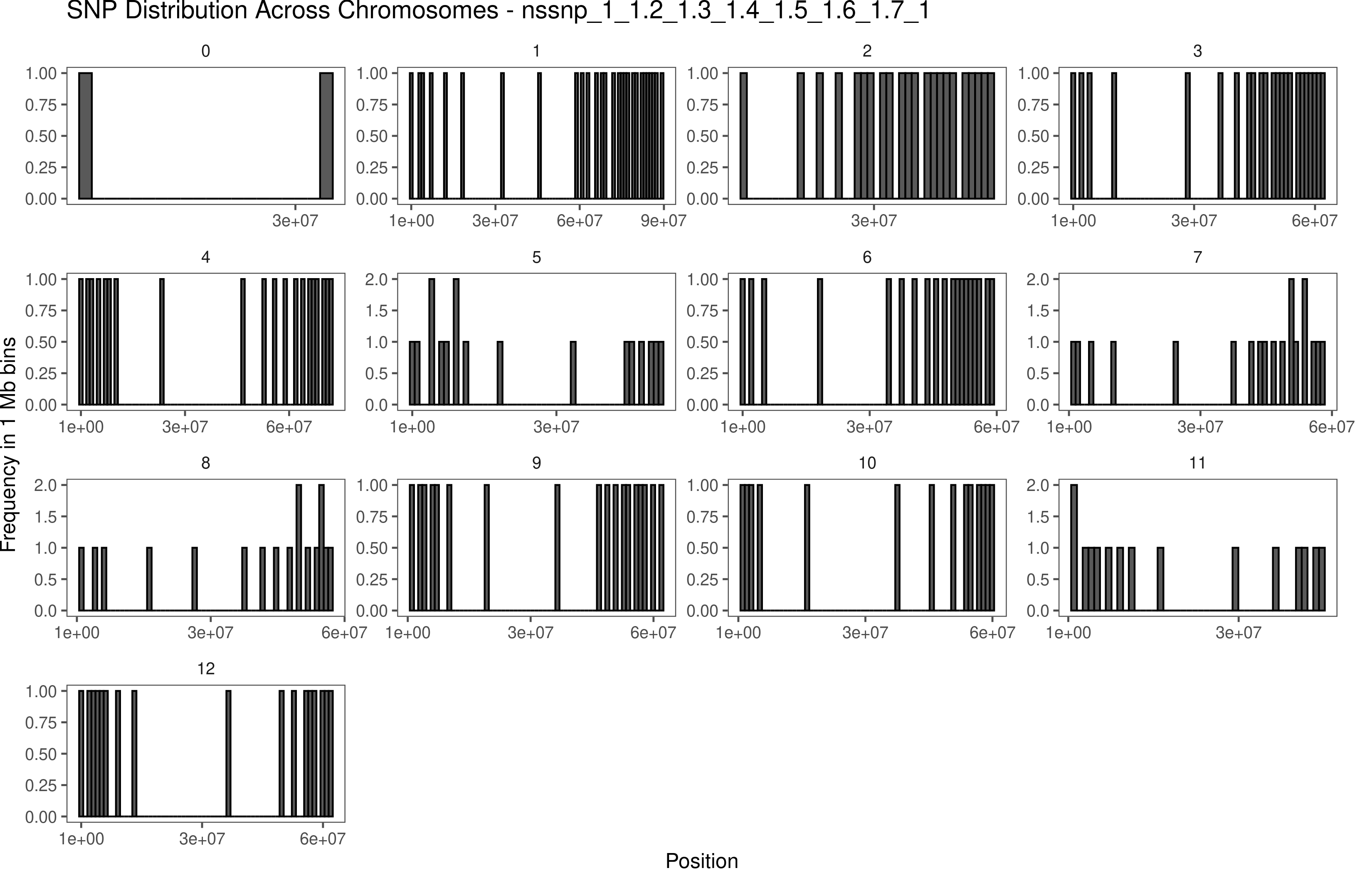


### Iteration 8


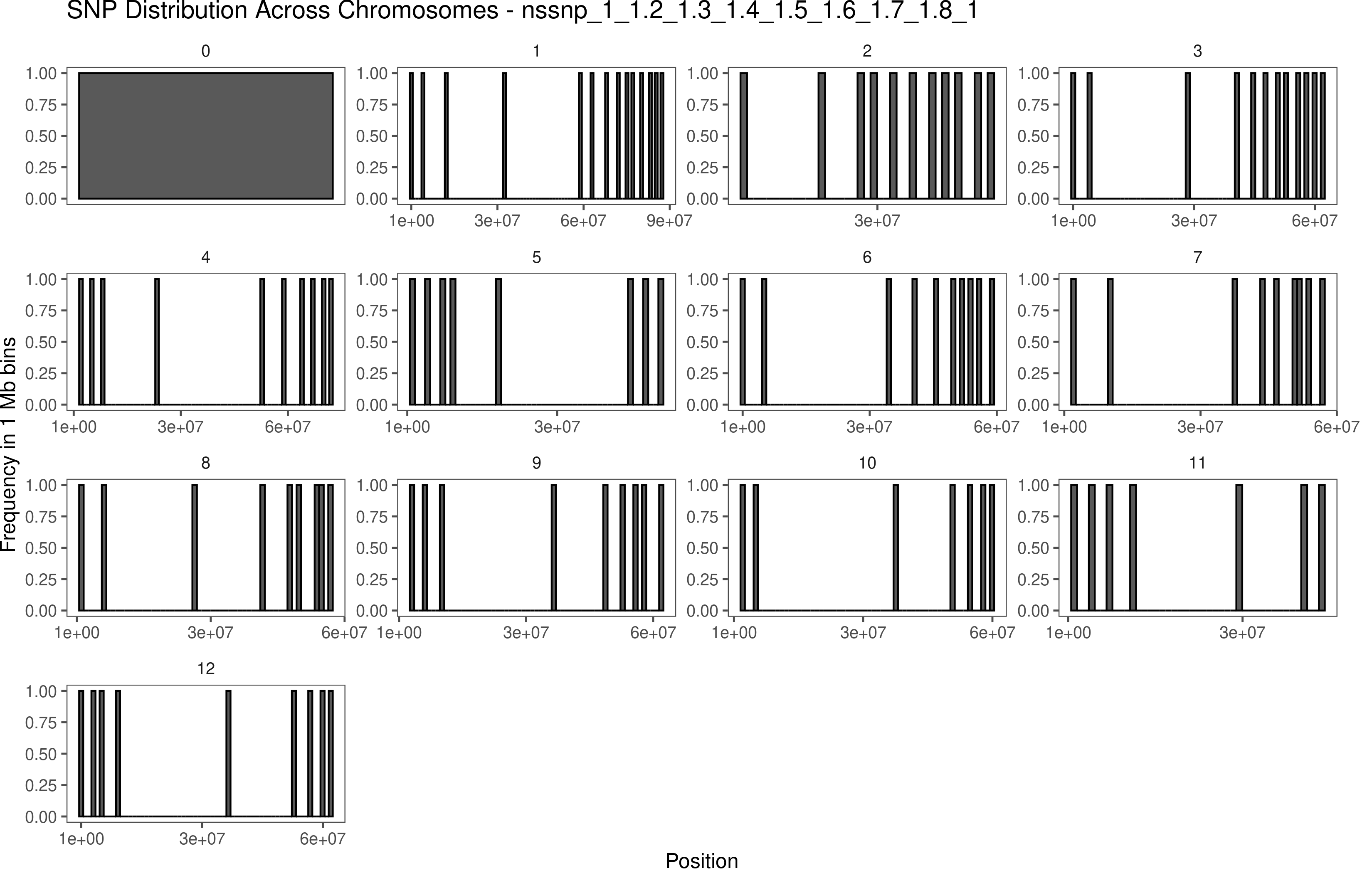


## Synonymous set

### Iteration 0 (complete set)


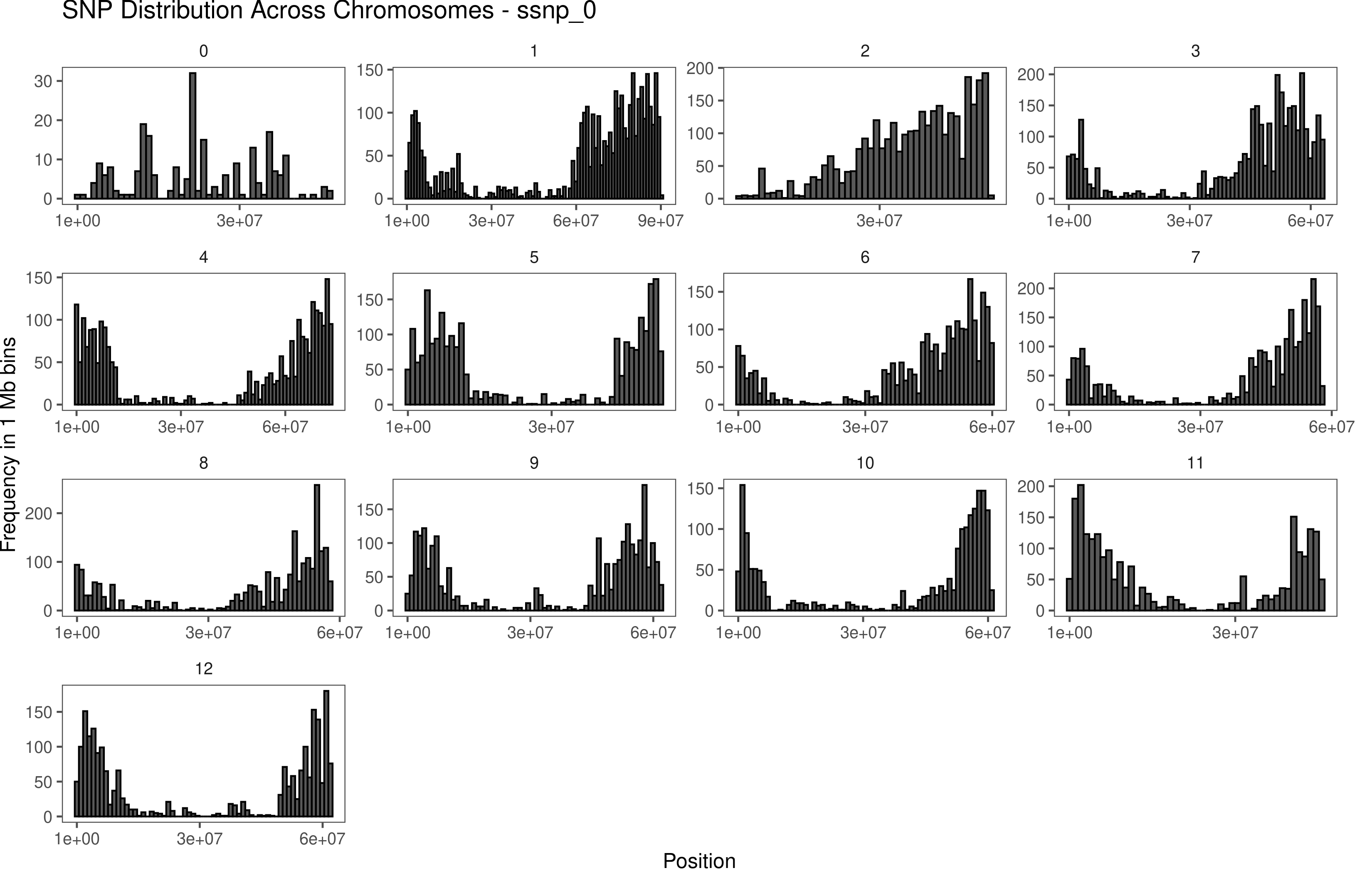


### Iteration 1


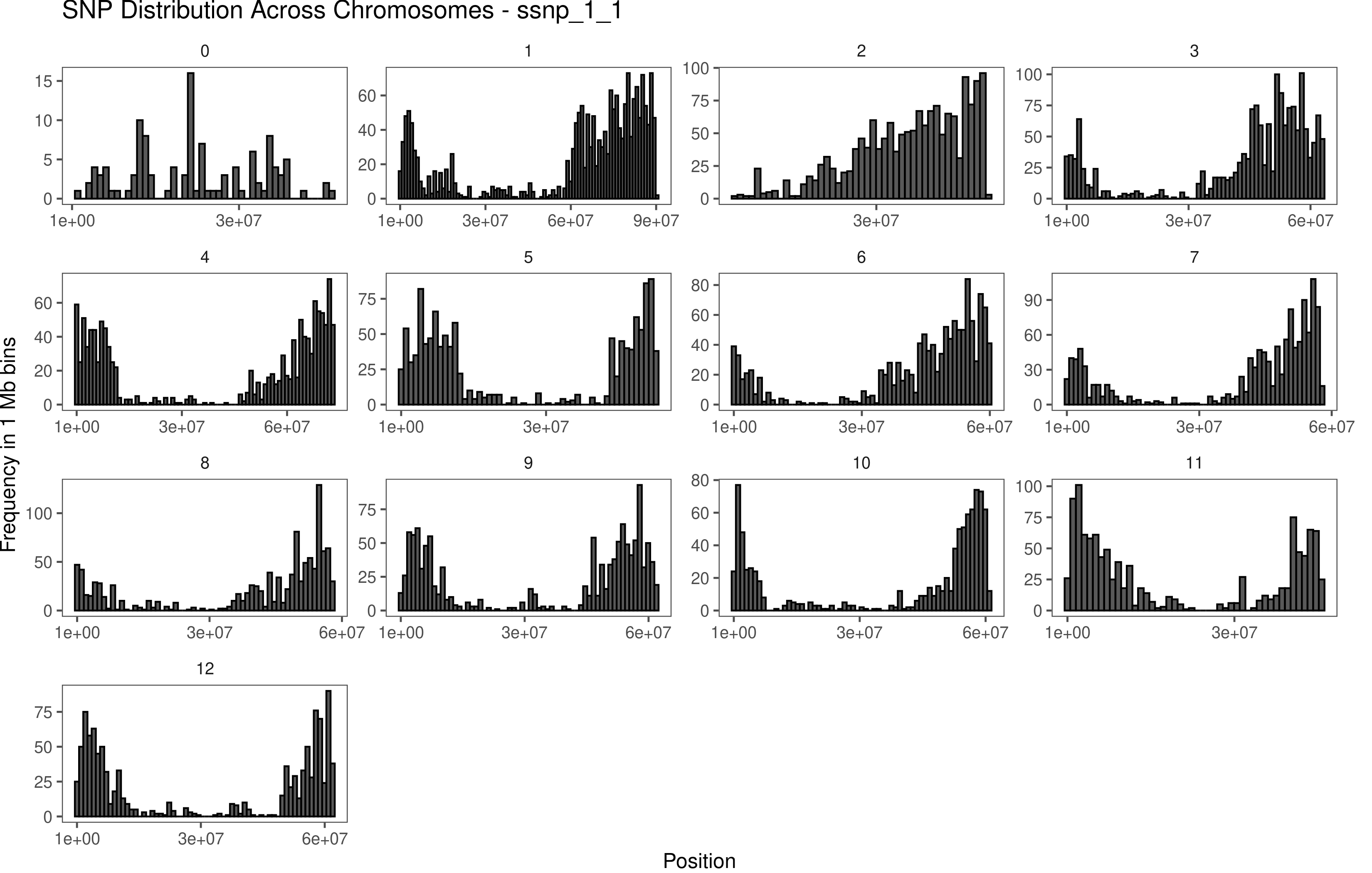


### Iteration 2


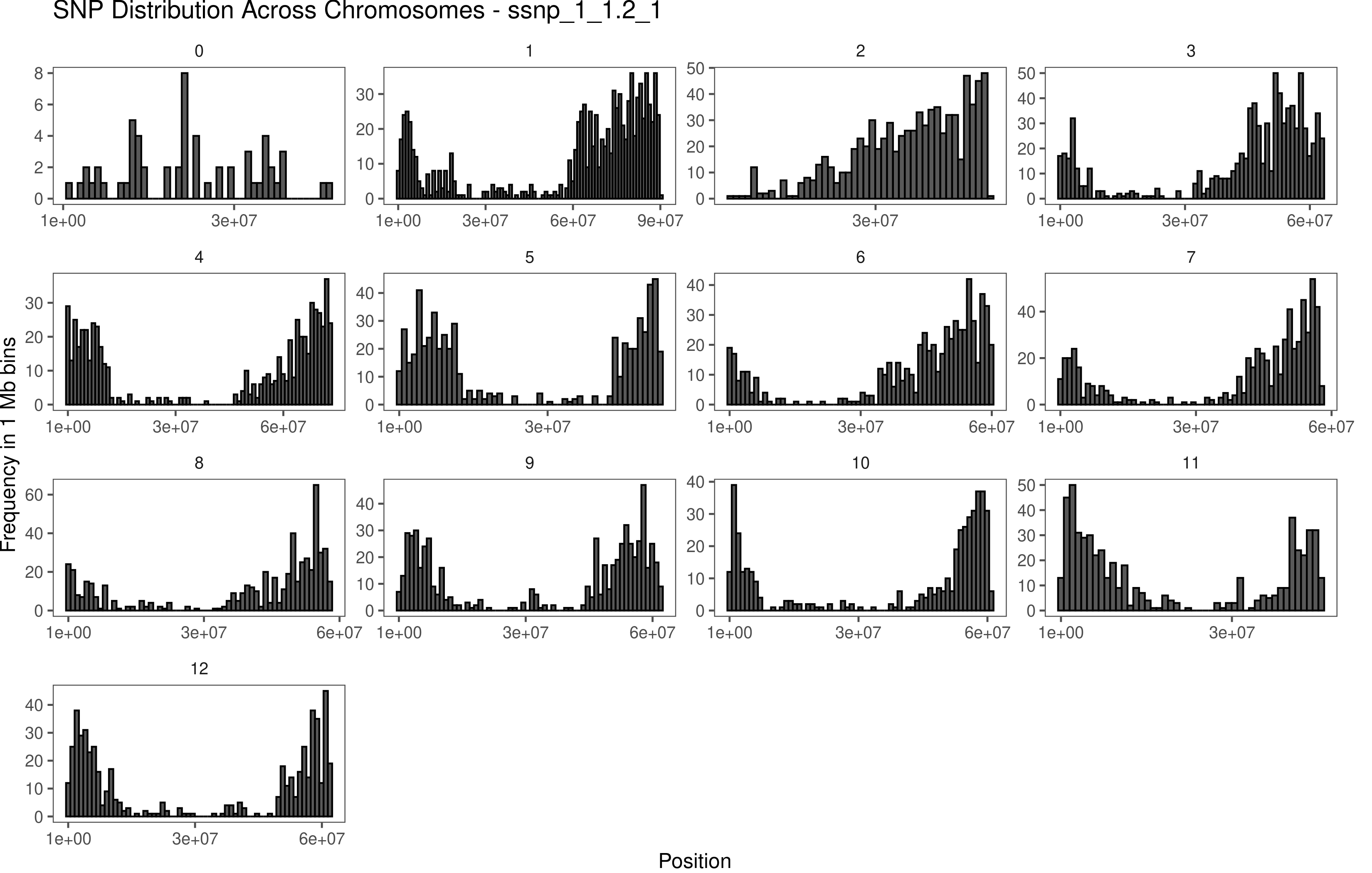


### Iteration 3


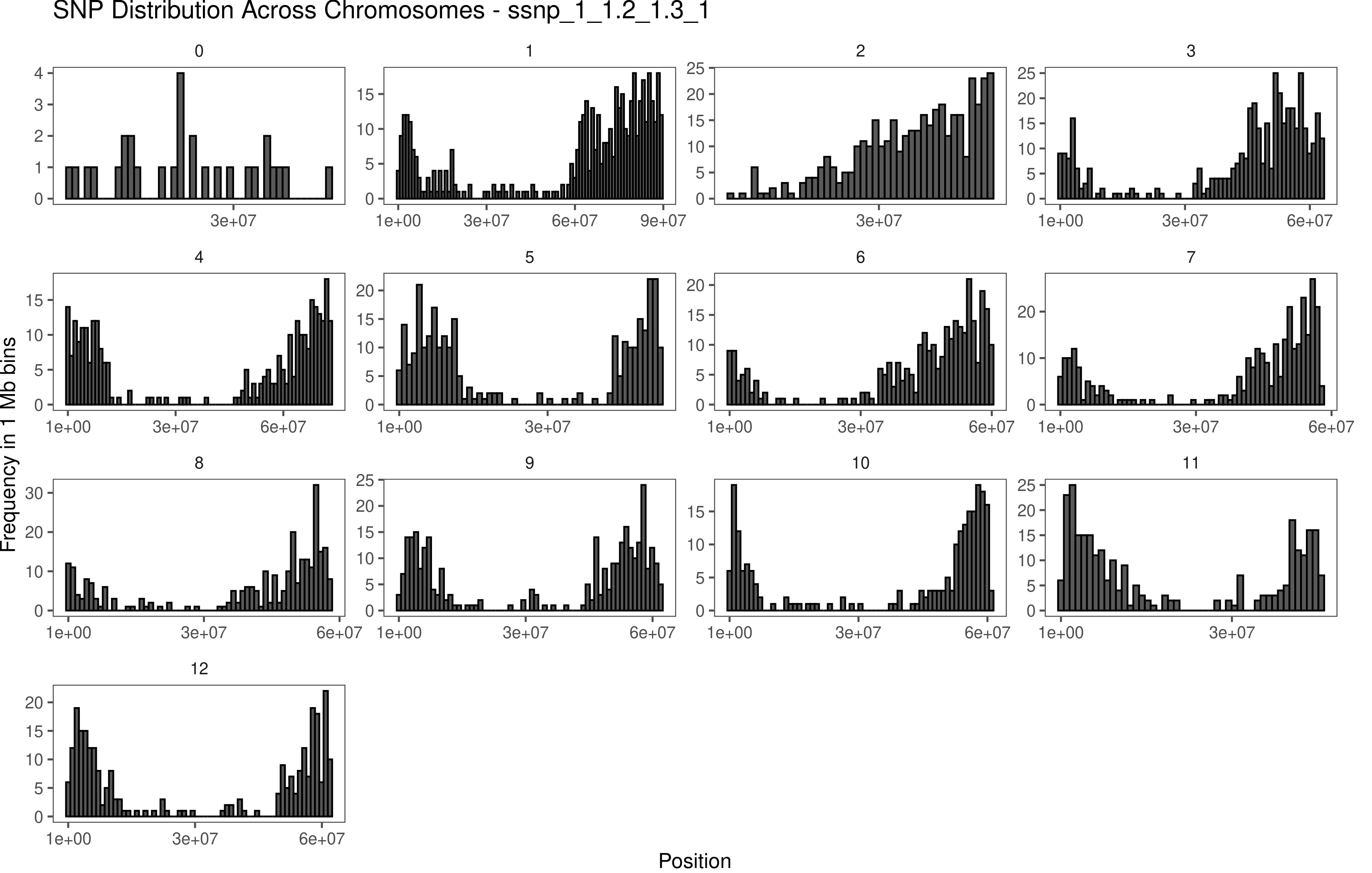


### Iteration 4


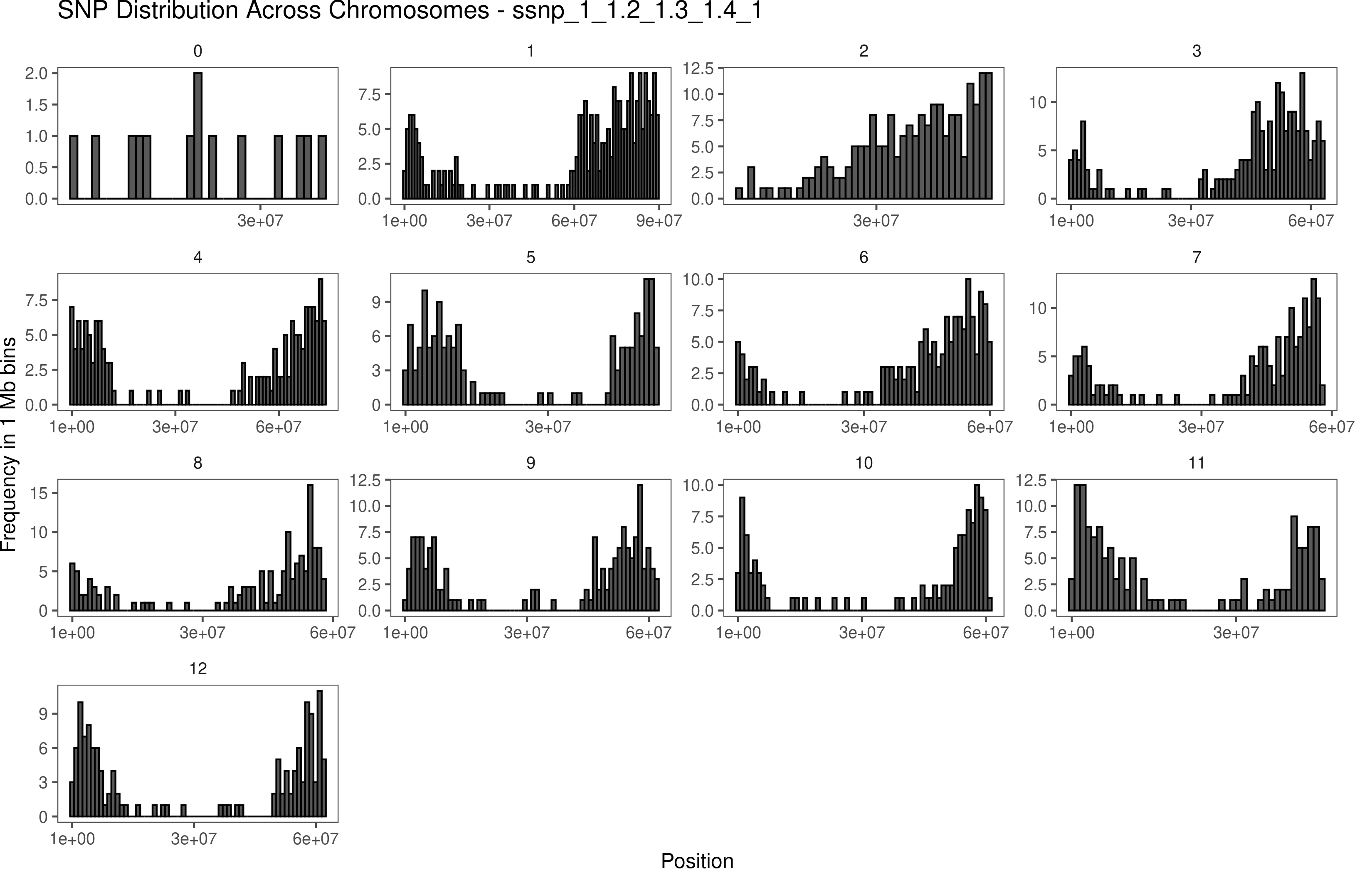


### Iteration 5


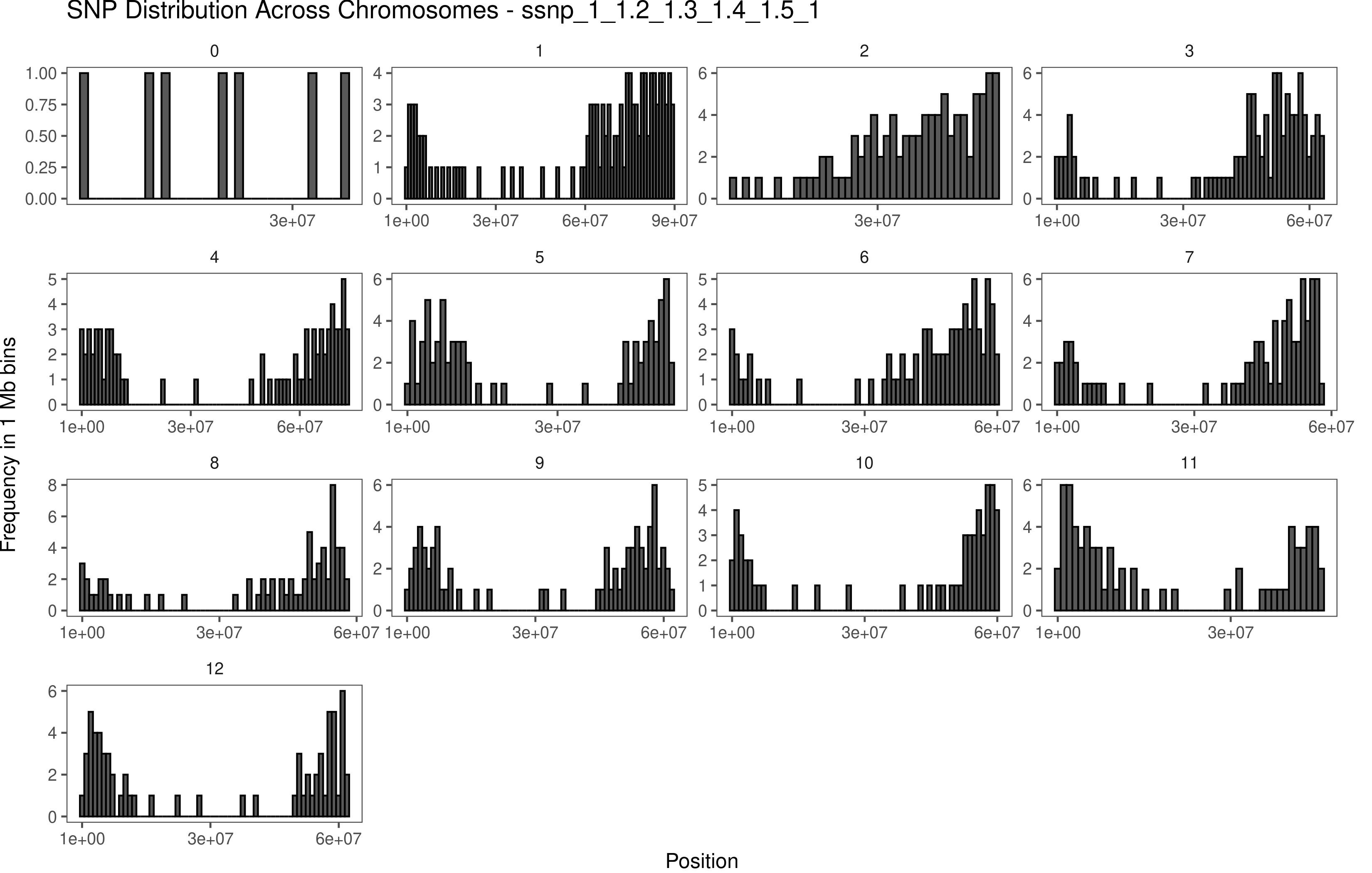


### Iteration 6


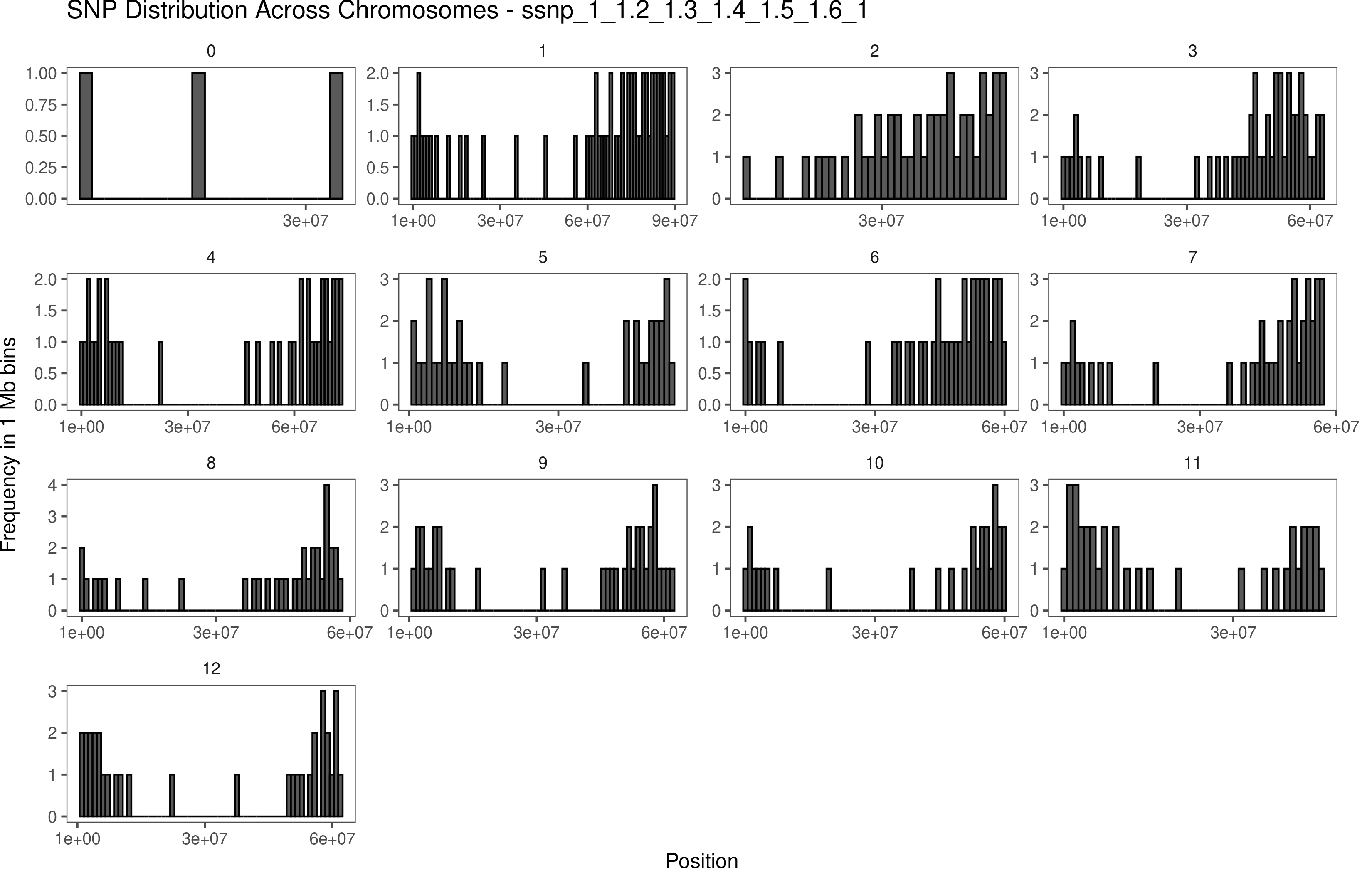


### Iteration 7


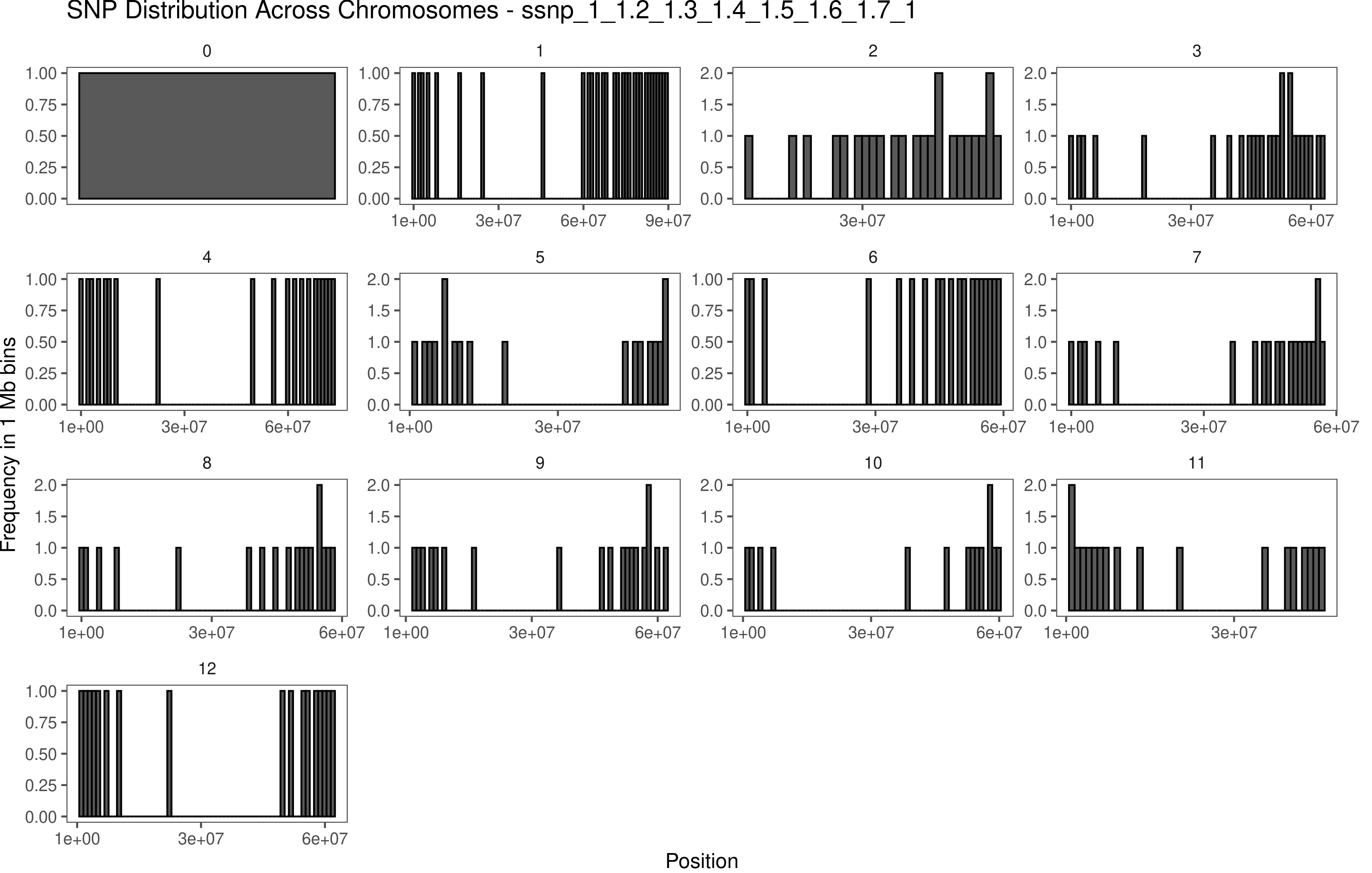


### Iteration 8


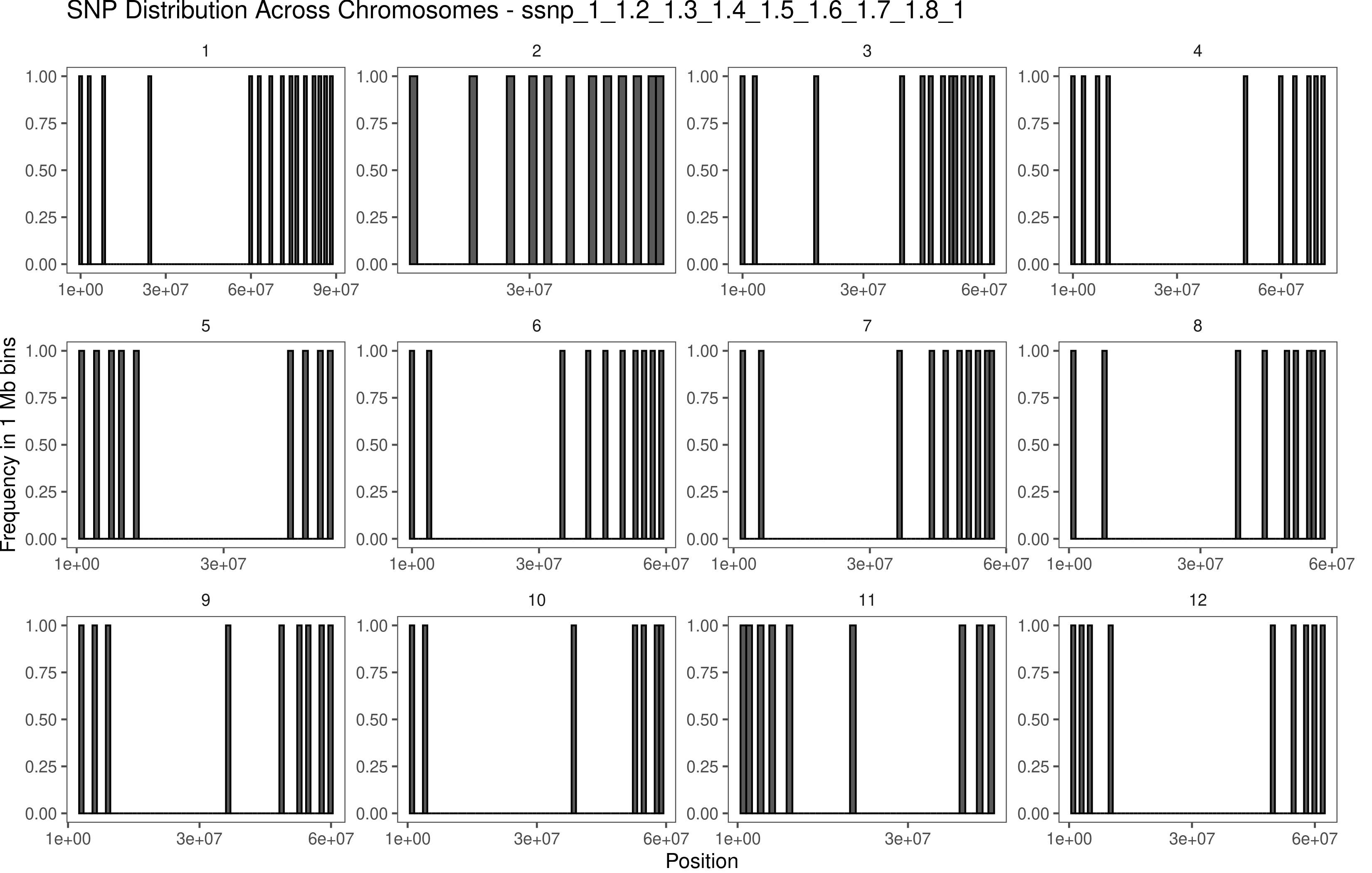


## Non-coding set

### Iteration 0 (complete set)


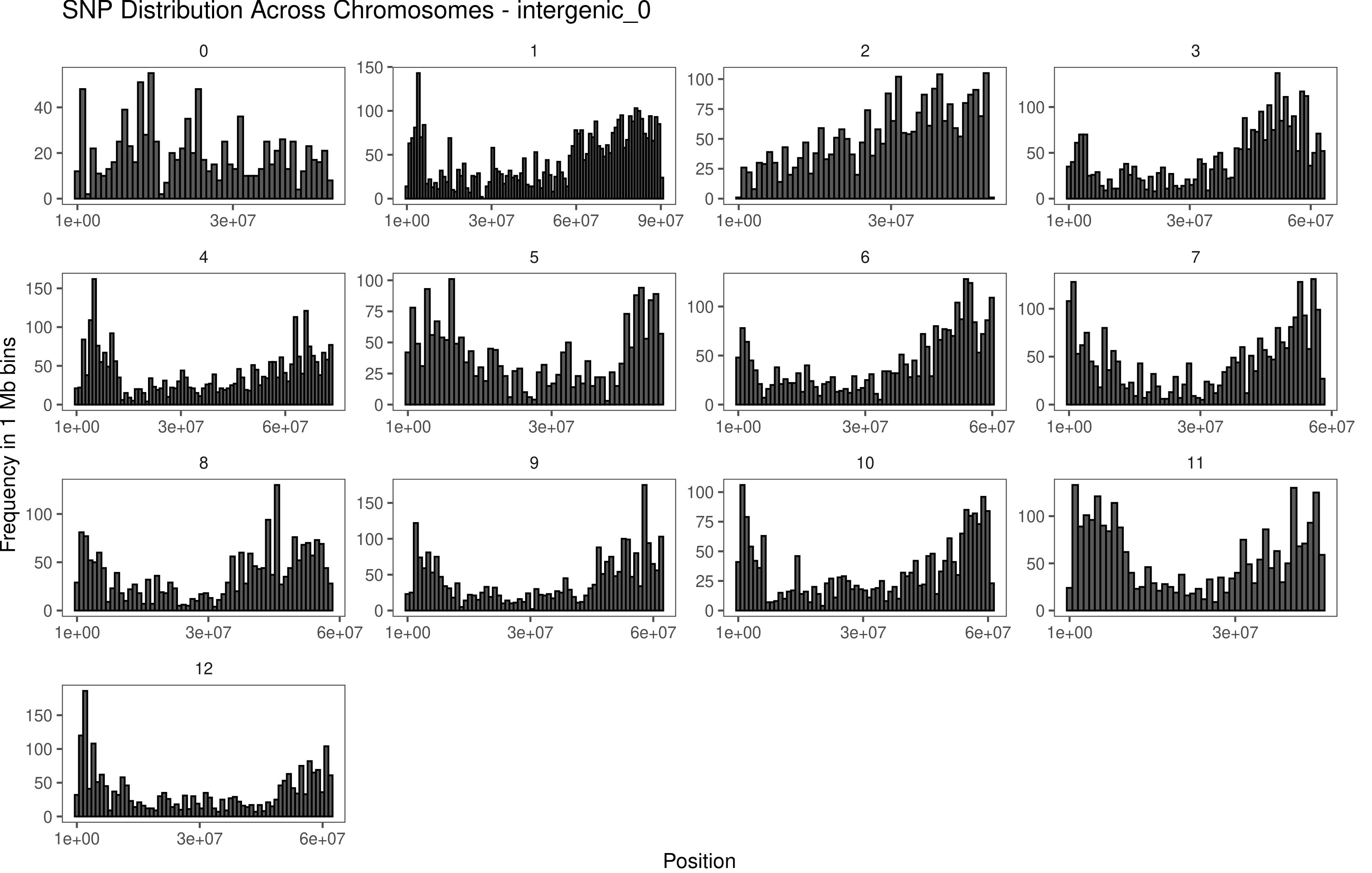


### Iteration 1


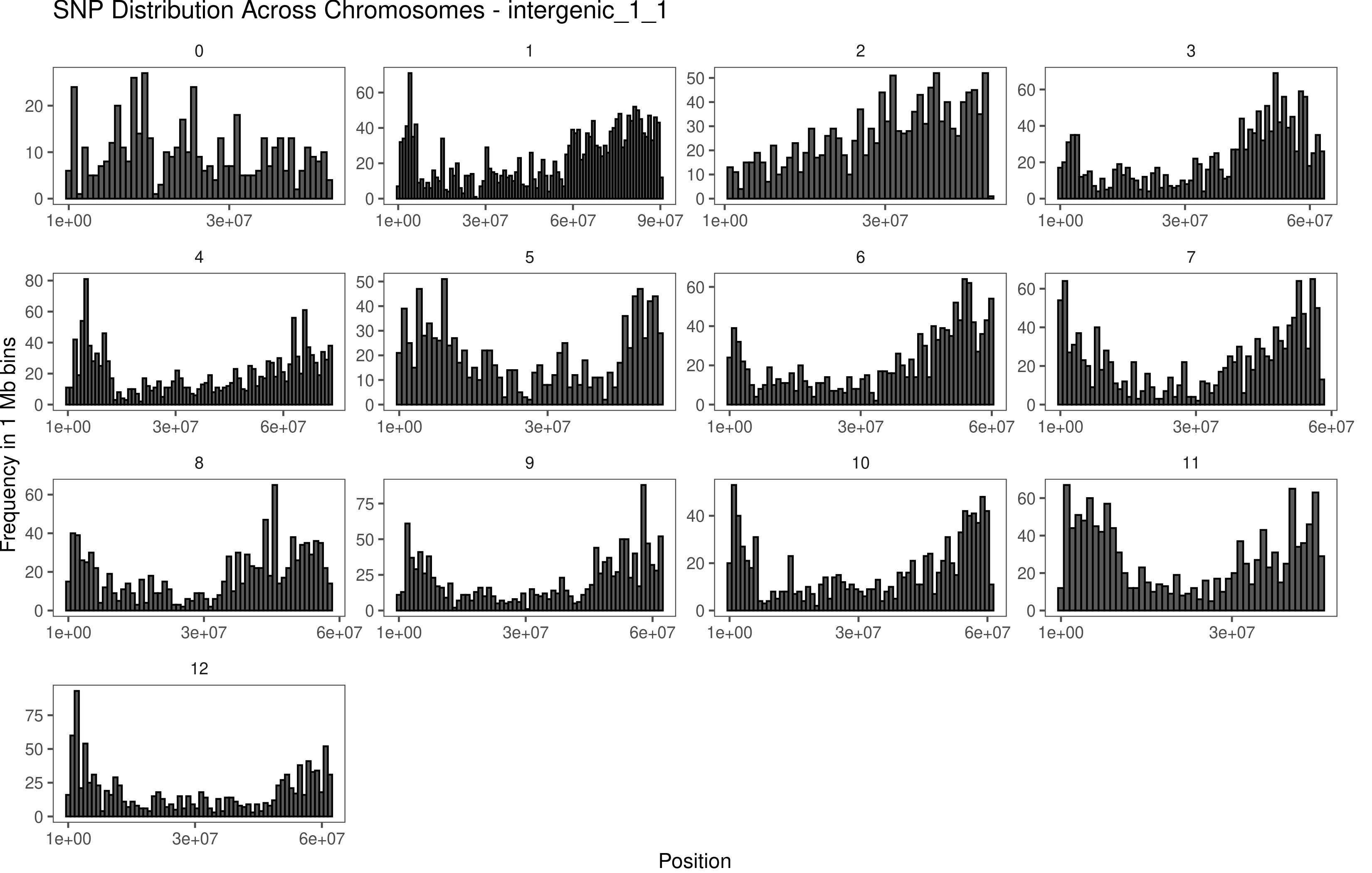


### Iteration 2


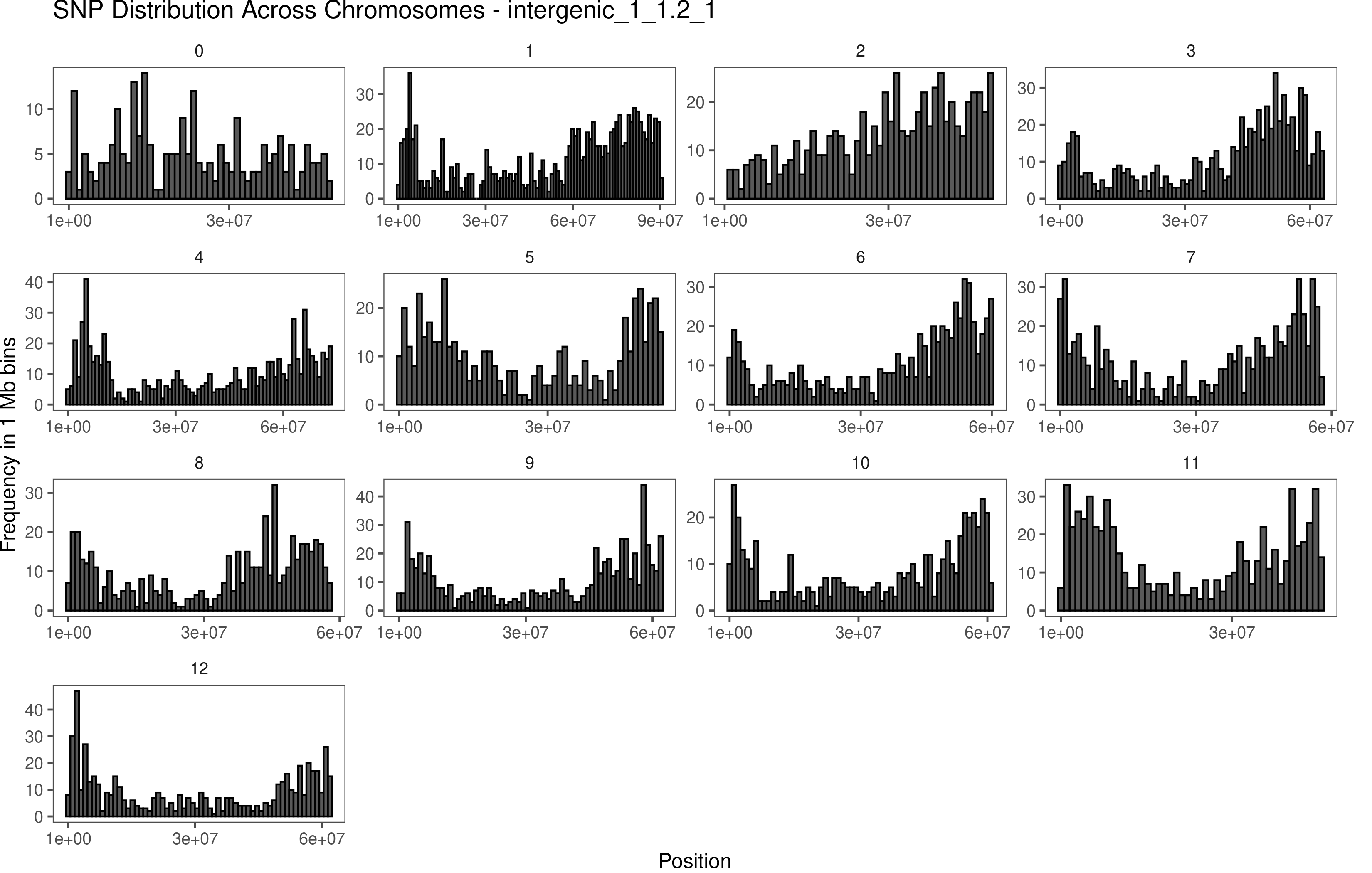


### Iteration 3


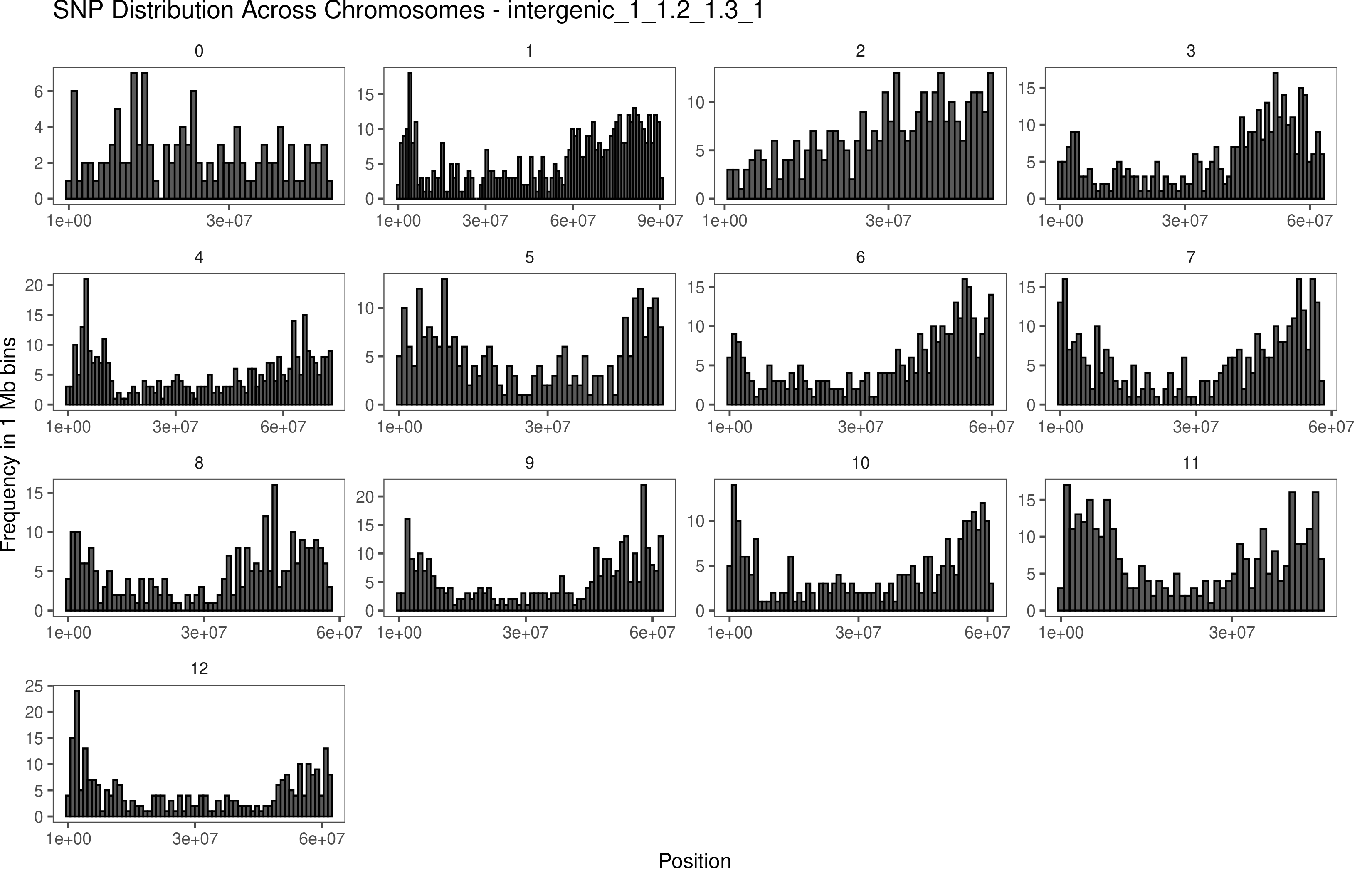


### Iteration 4


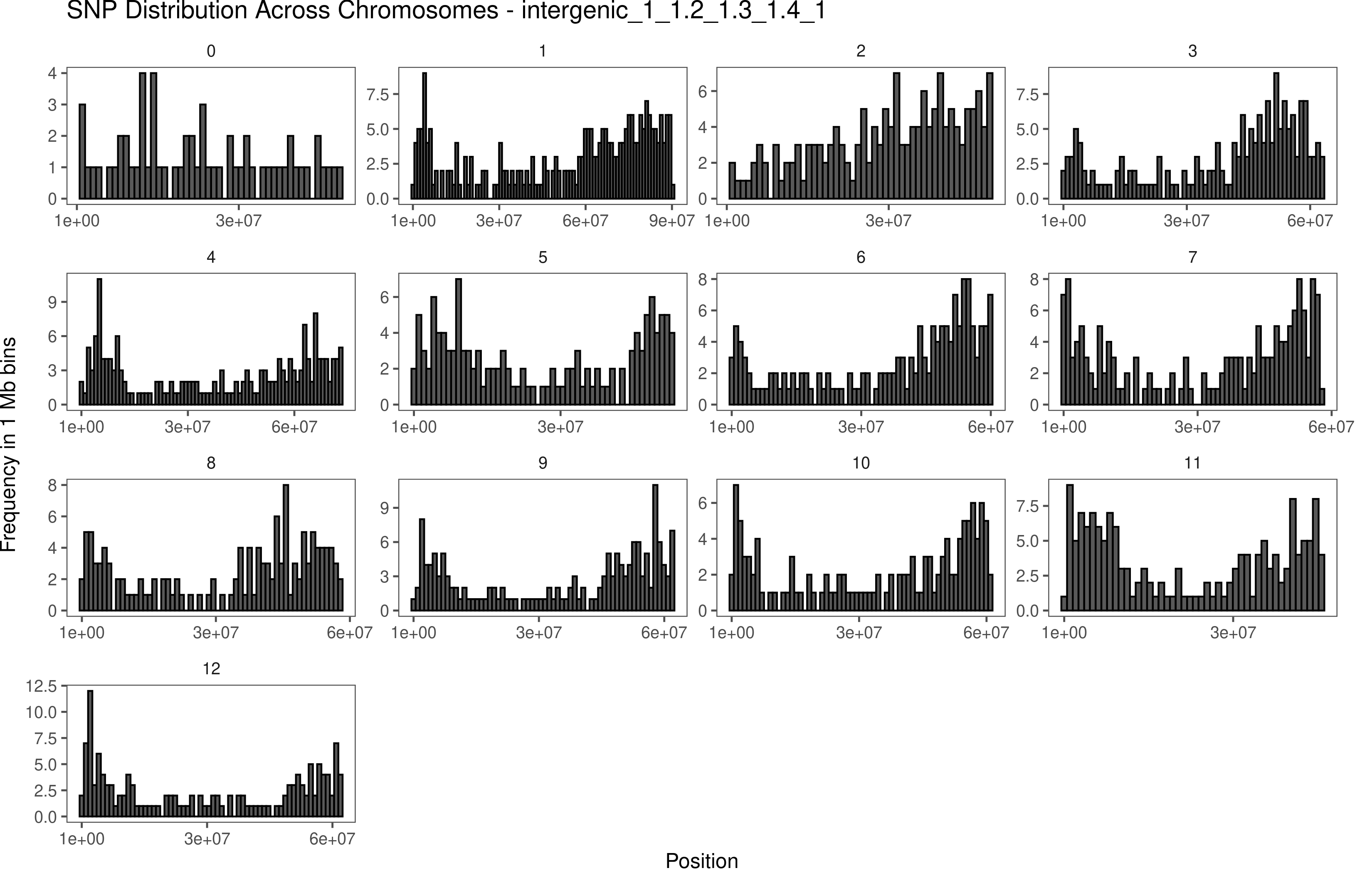


### Iteration 5


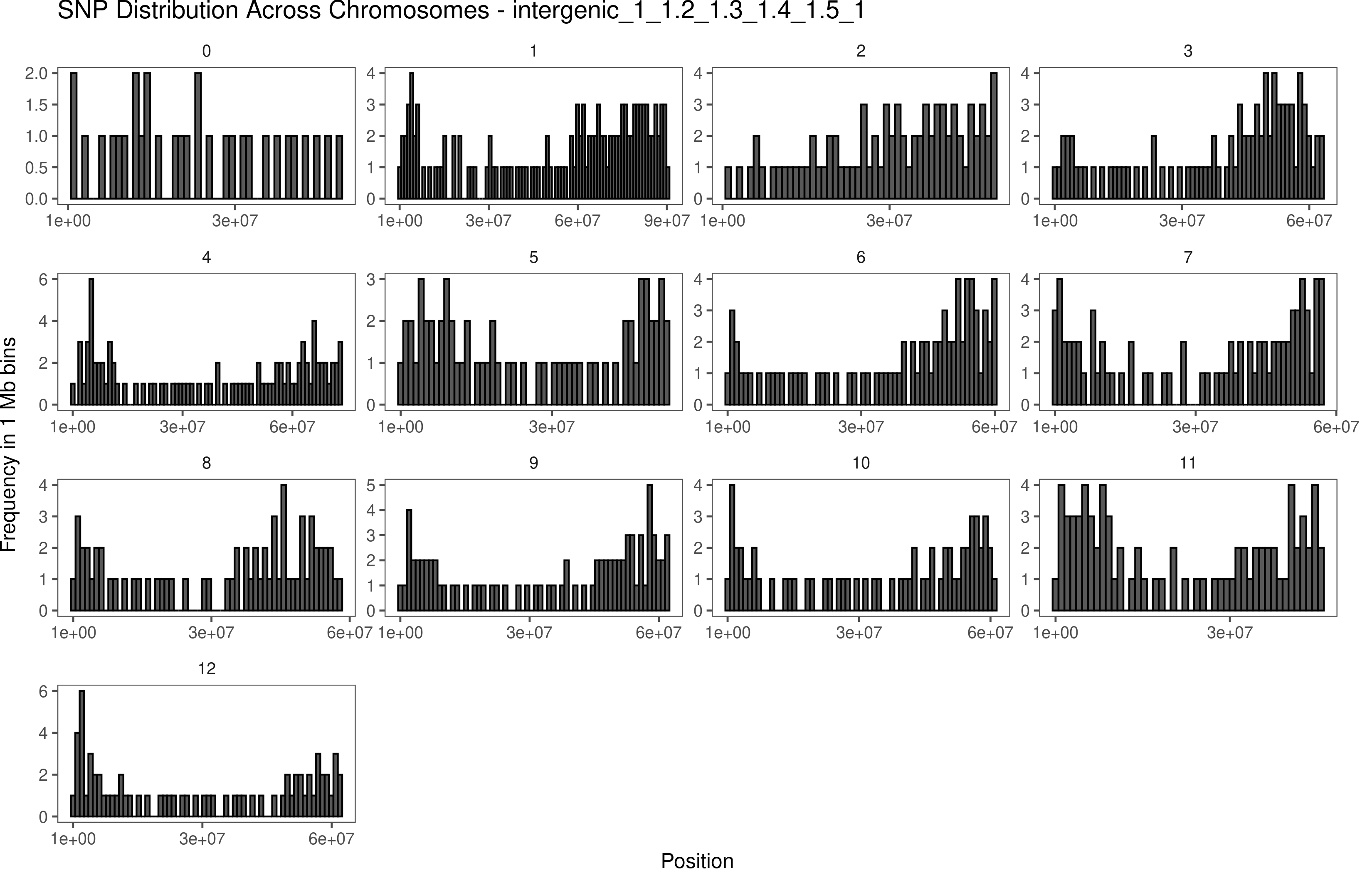


### Iteration 6


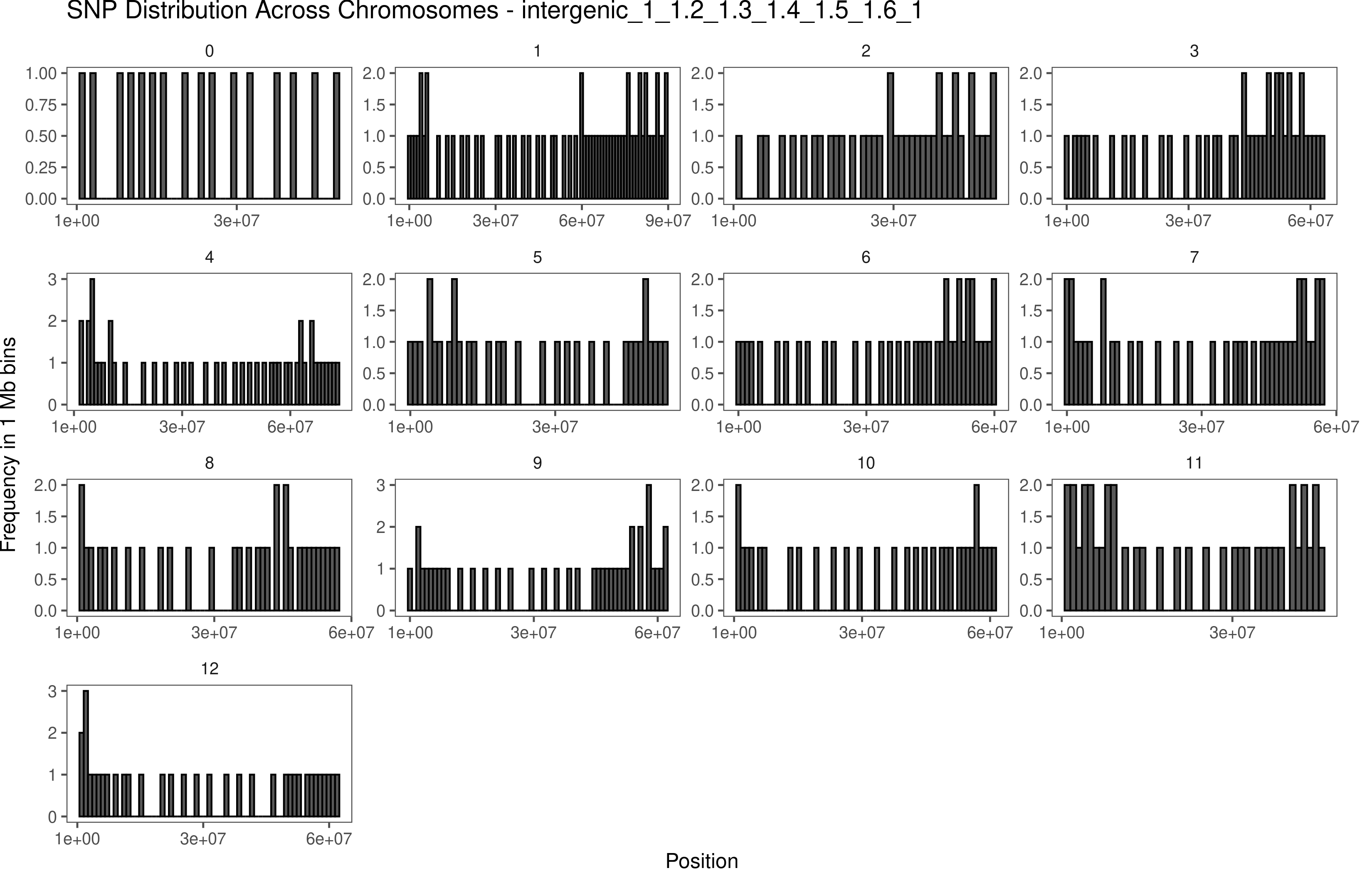


### Iteration 7


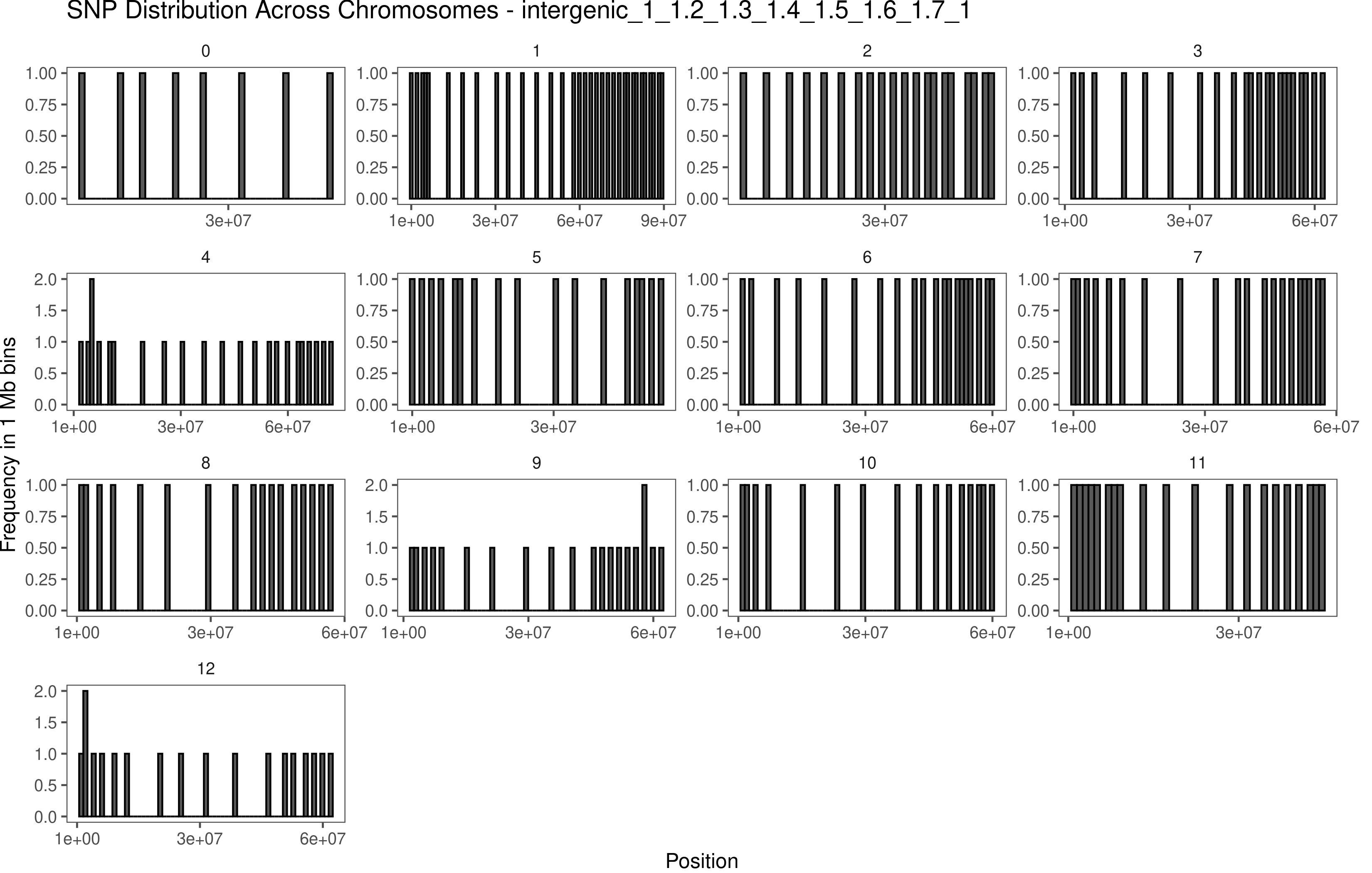


### Iteration 8


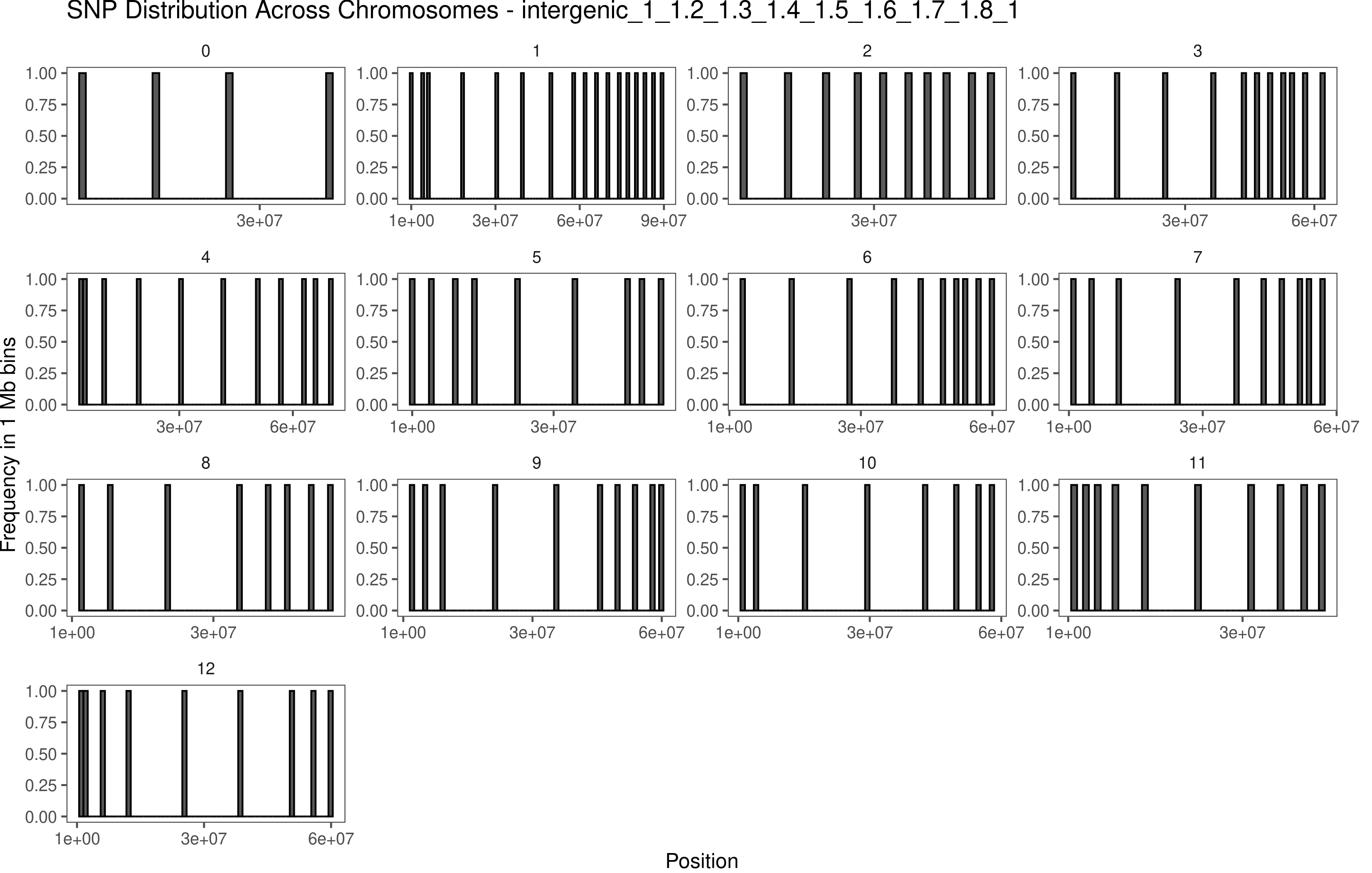

Supplement: Supplementary file 1 [file DataSheet_1.zip › SM File 5.docx]
